# Selective Monoreduction of 2,4 Diazido-Dideoxy Hexoses by Hydrogenation over Lindlar Catalyst

**DOI:** 10.1021/acs.joc.5c02433

**Published:** 2025-12-02

**Authors:** Leonie Wiener, Philipp J. Gritsch, Maximilian Kaiser, Nicolas Kratena, Peter Gärtner

**Affiliations:** Institute of Applied Synthetic Chemistry, TU Wien, A-1060 Wien, Austria

## Abstract

A novel, mild and site-selective hydrogenation of equatorial azides in diazido hexoses, achieved using Lindlar catalyst, is reported. Optimization studies on l-β-2,4-dideoxy-diazido rhamnoside revealed exclusive reduction of the equatorial azide while axially positioned azide groups remain untouched, affording the corresponding monoamino sugar in up to 87% yield. The methodology proved reliable across different diverse substitution patterns, consistently favoring equatorial over axial azide reduction while allowing for straightforward purification and scalability.

## Graphical abstract

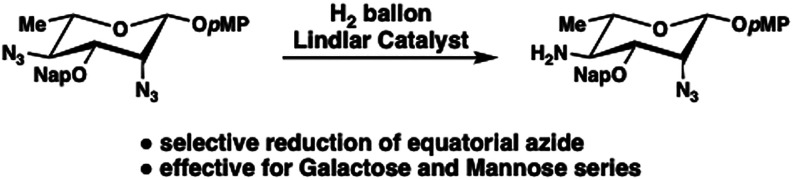

The selective modification of identical functional groups in a molecule continues to be one of the main ongoing challenges in synthetic organic chemistry. Especially the manipulation of carbohydrates requires chemists to discover methods which exploit minute differences in stereoelectronics of the hydroxy moieties on a sugar molecule. This has resulted in elegant contributions such as the tin-mediated selective derivatization of glycosides.^1^

In our research program we are interested in the synthesis and modification of hexoses decorated with at least one nitrogen functionality. These polyaminosugars, although rare, have been found in bacterial glycans such as the zwitterionic repeating unit from *Shigella sonnei* *O-*antigen (**1**, see Figure 1).^2^ These glycans feature a 2,4 diamino-fucose unit, which has also been found in other bacterial cell wall glycans, such as the lipoteichoic acid of *Streptococcus pneumoniae*.^3^ Bacterial nonulosonic acids, such as pseudaminic acid (**2**) from the periodontal pathogen *Tannerella forsythia*, show diverse substitution pattern on their amino functionalities and have been implicated in modulation of the immune system.^4^ Synthesis of such compounds have relied on nucleophilic additions to 2,4-diamino-2,4-dideoxy hexoses.^4b^ The biological activity of rare aminosugars is being leveraged, among others in the class of aminoglycoside antibiotics, highlighted here by isepamicin (**3**).^5^

**Figure 1:**
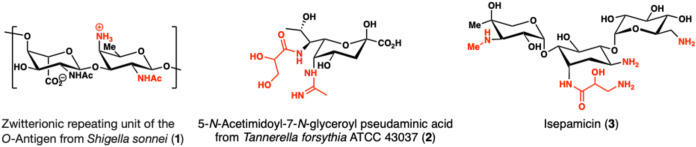
Diamino carbohydrate motifs in nature and in medicinal chemistry.

Syntheses of diamino carbohydrates typically rely on S_N_2 type reactions for the introduction of amino groups, often in masked form as either an azide or a phthalimide.^6^

The azide group exhibits excellent properties for the introduction of a nitrogen functionality in a multistep synthesis, its small size allowing for a facile and selective installation, before finally being unmasked at a convenient point to afford an amine. It is inert under a broad spectrum of reaction conditions but can be transformed, often orthogonally, into the desired amine via reduction, generally hydrogenation or using phosphor- or sulfur-based reagents.^6b,6c^

We were interested whether we could reduce selectively one azide of a 2,4-diazido hexose by leveraging the difference in reactivity of axial and equatorial azide substituents and form the corresponding amine in the process in a selective manner. Such a transformation would allow for the orthogonal introduction of amine substituents on hexoses, required for natural product synthesis and drug-development.^7^

In the literature, the site-selective reduction of polyazido glycans has been studied employing ruthenium nanoparticles,^8^ tetrathiomolybdate based reagents,^9^ thiol reagents^10^ as well as the Staudinger reduction employing trimethyl- and triphenylphosphine.^11^

We reasoned that this reaction could be, for the first time, accomplished by hydrogenation, given the right catalyst. This approach would offer regioselective functionalization of carbohydrates by orthogonal amine installation. Additionally, we envisioned a practical route with scalability and straightforward purification. L-β-2,4-dideoxy-diazido rhamnoside **6** was chosen for this investigation as the test substrate, as it had been used in literature for the synthesis of pseudaminic acid, a rare bacterial sugar decorated with two amino groups.^12^

## Synthesis of the Starting Material

Compound **6** was synthesized from 4-methoxybenzyl ß-L-fucopyranoside (**4)**,^12b^ by first selectively protecting OH-3 with a naphthylmethyl (Nap) group to give **5** (See Scheme 1). Treatment with triflic anhydride and subsequent substitution with tetrabutylammonium azide then revealed the desired 2,4-dideoxy-diazido-rhamnoside **6**.^6a^

**Scheme 1:**
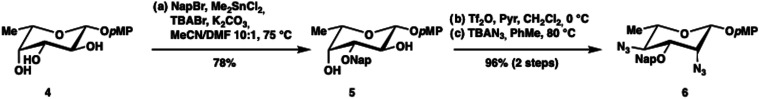
Starting Material Synthesis

## Reaction Optimization

We then set out to study the selective mono azide reduction of diazido-rhamnoside **6** under hydrogenation conditions (Table 1). The reaction was initially performed in methanol at room temperature with 5 mol % catalyst loading using a balloon filled with hydrogen gas until the reaction went to completion (TLC analysis). Filtration of the catalyst and removal of the solvent was followed by flash chromatography to reveal the reaction products. Analysis was accomplished by comparing ^13^C NMR shifts of C-2 and C-4 carbons in the starting material and the product (C–N_3_ = ca. 60 ppm, C-NH_2_ = ca. 50 ppm, see Supporting Information for HSQC spectra).

**Table 1:**
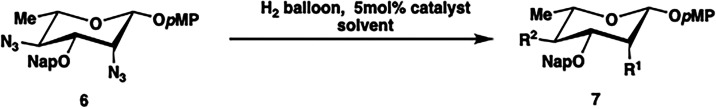
Reaction Optimization

entry	catalyst 5 mol %	solvent	result	yield [%]
1	Pd/C	MeOH [0.05M]	R^1,2^ = NH_2_	N/A^a^
2	Pd/BaSO_4_	MeOH [0.05M]	R^1,2^ = NH_2_, N_3_	N/A
3	Lindlar catalyst	MeOH [0.05M]	R^1^ = N_3_, R^2^ = NH_2_,	85
4	Lindlar catalyst	MeOH/Pyr 1:1 [0.2M]	R^1^ = N_3_, R^2^ = NH_2_	33^b^
5	Lindlar catalyst	MeOH/Pyr 1:1 [0.1M]	R^1^ = N_3_, R^2^ = NH_2_	65^b^
6	Lindlar catalyst	MeOH/Pyr 1:1 [0.05M]	R^1^ = N_3_, R^2^ = NH_2_	87

a Nap group was cleaved.

b Starting material recovered.

The first catalyst investigated was palladium on charcoal (**entry 1**). Unsurprisingly, the sole product of this reaction was a 2,4-diaminorhamnoside where no azide remained. In addition the Nap group was cleaved under these conditions. Subsequently we turned to catalysts with reduced reactivity, such as the Rosenmund catalyst, palladium on BaSO_4_ (**entry 2**). After full conversion of the starting material, three products were isolated: a 1:1 mix of monoreduced compounds, where the azide at either C-2 or C-4 was reduced as well as the direduced 2,4-diaminorhamnoside. This result showed a stepwise reduction process, however no discrimination of axial or equatorial substituent as well as overreduction to the diamine. We then turned to the Lindlar catalyst, palladium on calcium carbonate, poisoned with lead (**entry 3**) for the hydrogenation. With this catalyst only a single compound was isolated in 85% yield: the desired 2-azido-4-amino rhamnoside **7**. This result showed selective mono reduction of the equatorial azide at C-4 under hydrogenation conditions.

When investigating the reaction, the low solubility of **6** in methanol became apparent: dissolution of the substrate was only achieved at concentrations of 0.05 M or lower. We then investigated other solvents, but neither ethyl acetate, ethanol nor tetrahydrofuran showed any improvement. Using pyridine as a cosolvent in a 1:1 mixture with methanol greatly improved the dissolution. However, when the reaction was conducted at higher concentration of 0.2 M (entry 4) the reaction stalled after some time and incomplete consumption of starting material was observed. Decreasing the concentration improved and the yield and conversion, however it remained incomplete (entry 5). To achieve complete conversion and improved yield, a concentration of 0.05 M had to be used for optimal reaction performance (entry 6).

These optimized conditions were used from this point on, as they delivered product **7** in a slightly increased yield of 87% in a reproducible manner without solubility issues. More importantly, showcasing that this is not an isolated optimized example, other substrates which were investigated as part of this study (see Table 2), were even less soluble in pure methanol but were sparingly soluble in the new 1:1 mixture.

The reaction optimization studies were performed using 0.1 mmol of substrate showing a reaction that required minimal purification. Scale-up of this reaction also proved to be straightforward, at 10 mmol scale the reaction essentially proceeded the same way with a minimal decrease in yield (Scheme 2). Additionally, the crude product obtained after removal of the catalyst was generally pure enough to perform subsequent reactions such as Troc protection of the resulting amine in 82% yield over two steps. This highlights the high selectivity and mild nature of the reaction, creating essentially only a single product.

**Scheme 2:**
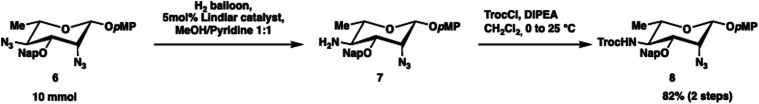
Synthesis of Troc-Protected Rhamnoside

The highly selective reduction of the equatorial azide in diazido rhamnoside **6** prompted us to investigate the broader applicability of this reaction. As depicted in Table 2, the selectivity of this system toward the equatorial azide remains, regardless of whether it is attached to the C-2 or C-4 of the carbohydrate.

**Table 2:**
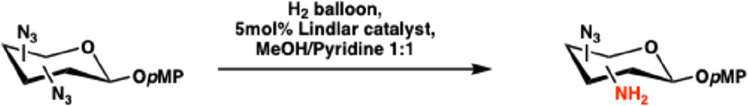

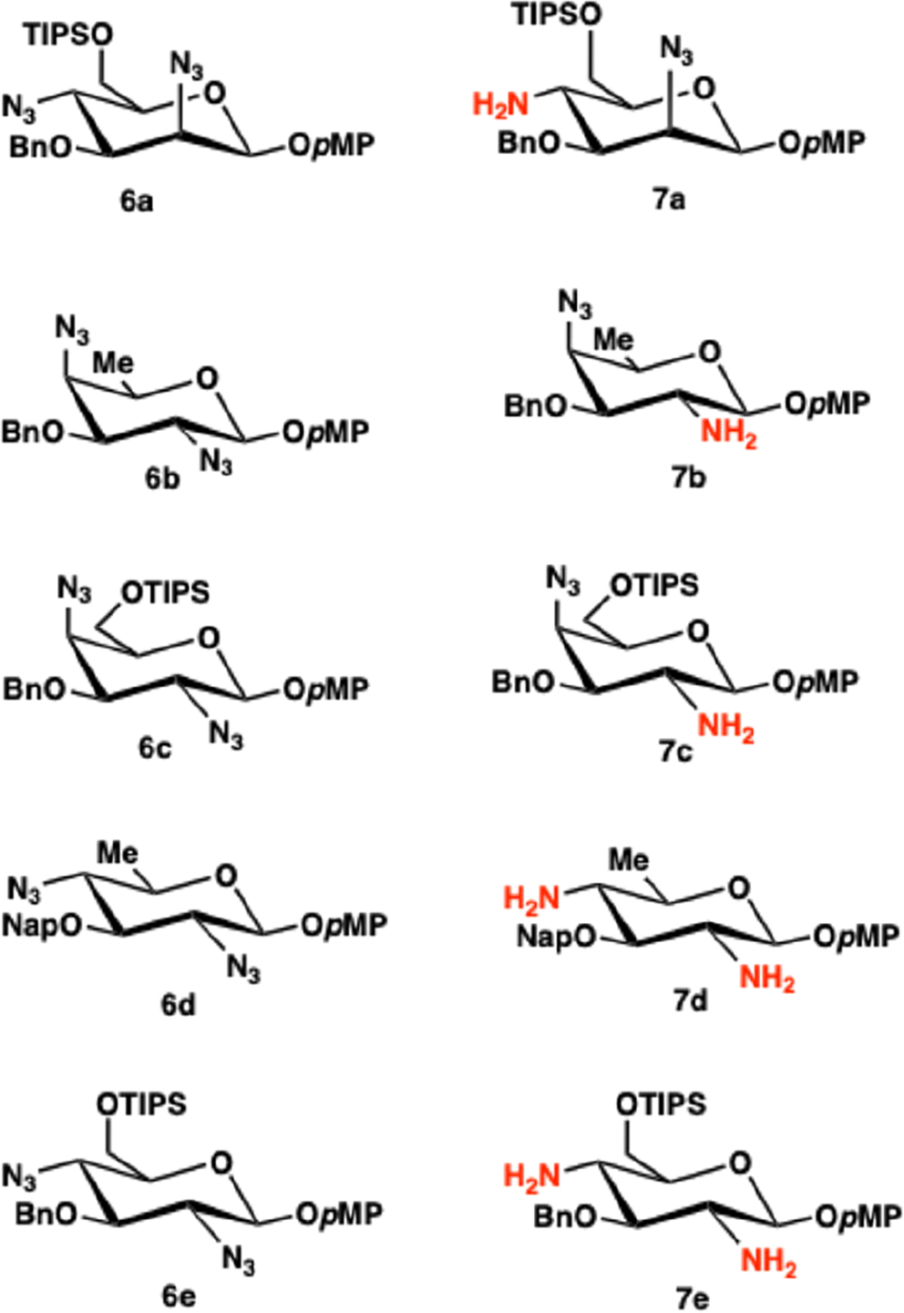
Selective Reduction of Equatorial Azides

Entry 1 shows diazido mannoside **6a**, which in comparison to **6**, is additionally decorated with a triisopropylsilyl group at the C-6 position. Pleasingly this additional group has no impact on the selectivity or efficiency of the reaction. Entries 2 shows diazido fucoside **6b**, where the equatorial and axial positions are switched compared to **6**. Again, the equatorial azide is reduced, and no other product was observed, similarly to entry 3, where diazido galactoside **6c** was investigated. The yield of the reaction remains above 70% throughout the compounds screened.

Entries 4 and 5 show substrates with two equatorial azide groups and it was expected that both azides would be reduced. To our surprise, we found that under the investigated conditions, no product was isolated and only starting material was recovered in 90–95% yield. We also intended to investigate substrates where both azides are axially positioned, however we were unable to synthesize these compounds according to the general procedure described in Scheme 1. During our studies, the introduction of both azides *via* S_N_2 reaction would stall after the first transformation. Even after elevated temperatures only monoazide triflyl compounds were recovered.

In general, only a handful of examples have been reported on the selective reduction of polyazido-glycosides. The selective reduction of either axial or equatorial azide hexose substituents has only once been the focus of a study: Li et al. investigated the regioselective reduction of 2,3 and 2,4 diazido hexopyranosides with triphenylphosphine.^11e^ Generally, the conversion of azides to amines by hydrogenation has been considered to be a nonselective process in the literature.^9,13^

Nevertheless, the methodology presented herein achieves excellent site selectivities in the hydrogenation of 2,4 dideoxy diazido β-pyranosides. The sole product isolated displays the equatorial azide reduced, independent of its attachment to either C-2 or C-4.

Li et al. found as well that equatorial azides were more susceptible to reduction than axial azides, but that α-pyranosides usually displayed better selectivities than β-pyranosides.^11e^ The authors investigated whether stereoelectronic factors contributed to the selectivity observed, which had been discussed by the groups of Wong and Chang,^11a–11d^ but did not find evidence for it. In contrast, they stated that the selectivity observed was mainly attributed to steric hindrance and concluded that 1,3 diaxial interactions were destabilizing the reaction between the axial azide and the incoming reagent, leading to the observed selectivity.

Steric accessibility is thus assumed to be the main driving force for the selectivity observed in the hydrogenation reaction reported in this work. The quasi-flat surface of the loaded catalyst strongly discriminates the different faces of the sugar molecule allowing only minimal repulsion for a successful delivery of hydrogen. The difference in electronic activation of each azide, as shown by the ^1^H NMR shift of *ipso*-protons, is of little influence on the reaction outcome. In contrast, the interaction of the equatorial azide on the surface of the catalyst is likely energetically favored over the axial azide.

Unexpectedly, no hydrogenated products were obtained when substrates **6d** or **6e** were subjected to the optimized conditions; instead, 90–95% of the starting material was recovered.(Table 2, **entries 4 and 5** respectively) This may be explained by the observation that the hydrogenation of **6** would stall at higher concentrations (see Table 1 entries 3–6) which would be consistent with catalyst inhibition, a common issue in palladium-catalyzed transformations of biomolecules.^14^ We propose that the amine functionality generated upon initial reduction coordinates strongly to the Lewis-acidic palladium surface, thereby rendering the catalyst inactive. At lower concentrations (Table 1, entry 6), increased solubility of the product alleviates this inhibition, allowing catalyst regeneration and improved conversion. In the case of **6d** and **6e**, reduction of both azides furnishes a diaminohexose that binds so tightly to palladium that dissociation is no longer feasible, effectively deactivating the catalyst after a single turnover. The recovery of the starting material in 90–95% further supports this scenario, suggesting that partial conversion followed by strong product–catalyst complexation prevents subsequent hydrogenation. Efforts to overcome this limitation and enhance conversion are currently underway.

In summary we report for the first time a mild and neutral method to selectively hydrogenate equatorial over axial azides on hexoses mediated by the Lindlar catalyst. The distinguishing characteristic of this methodology is its high selectivity, ease of purification and scalability. Further studies based on this work are currently in progress.

## Experimental Section

### General Methods

Unless stated otherwise, all chemicals were purchased from commercial suppliers (Sigma-Aldrich,BLDPharm, TCI, abcr, Acros, Fisher, VWR, FluoroChem, Angene) and used without further purification. Lindlar catalyst was purchased from TCI, Product. No 1703: Palladium 5% on Calcium Carbonate (poisoned with Lead). Some dry solvents (toluene, CH_2_Cl_2_, THF, Et_2_O) were obtained from a PureSolv SPS system by Innovative Technologies. Dry MeCN, pyridine, and all other dry solvents were obtained from Acros Organics over molecular sieves and used without further purification. All other solvents used were HPLC grade or p.a. unless stated otherwise. Reactions were carried out in round-bottom flasks, oven-dried Schlenk flasks or microwave vials under an inert atmosphere (Argon) unless stated otherwise. Unless stated otherwise, reactions were carried out at 25 °C. Reactions that required heating were heated with an oilbath with a temperature probe inserted into the oil for temperature control. ^1^H, and ^13^C NMR spectra were recorded on a Bruker AVIII 400 Spectrometer (^1^H: 400 MHz and ^13^C: 101 MHz) or a Bruker Avance III 600 (^1^H: 600 MHz and ^13^C: 151 MHz) in, CDCl_3_ or CD_3_OD and referenced to residual solvent peaks. Chemical shifts δ are quoted in parts per million (ppm) to the nearest 0.01 for ^1^H and 0.1 for ^13^C, coupling constants J are quoted in Hz to the nearest

0.1 and splitting are recorded as singlet (s), doublet (d), triplet (t), doublets of doublets (dd), doublets of doublets of doublets (ddd), doublets of triples (dt). Assignments were based upon COSY, HSQC and HMBC experiments. Any grease or residual solvent impurity will be indicated in the spectrum. Analytical thin layer chromatography was performed on precoated silica gel aluminum sheets from Merck (TLC Silica Gel 60 F254). Spots were visualized either by the quenching of UV fluorescence or by staining with phosphomolybdic acid/cerium sulfate, or acidic p-anisaldehyde or vanillin solutions. Preparative column chromatography was carried out using Geduran Silica Gel 60 (40–63 μm) from Merck or LiChroprep RP-18 (25–40 μm), which will be indicated as “fine silica”. In cases where mixtures of solvents were used, the ratios refer to the component volumes. In cases where gradients where used, the start and the end ratio are stated. The HR-MS analysis was carried out from methanol or acetonitrile or water or a mixture of these solvents (concentration: 10 μM) by using an Agilent G7167B multi sampler, an Agilent G7120A binary pump with degasser, an Agilent G7116B oven and Agilent 6545 Q-TOF mass spectrometer equipped with a dual AJS ion score.

#### (4-Methoxy)­phenyl 3-O-(2-naphthylmethyl)-β-L-fucopyranoside (**5**)

To a stirred solution of (4-Methoxyphenyl)- β-L-fucopyranoside (**4**)^15^ (17.65 g, 43.0 mmol, 1.0 equiv) in 220 mL () of a 10:1 mix of MeCN and DMF was added potassium carbonate (6.49 g, 64.5 mmol, 1.5 equiv), tetrabutylammonium bromide (0.90 g, 4.3 mmol, 0.1 equiv) and dimethyltin dichloride (0.95 g, 4.3 mmol, 0.1 equiv). This suspension was heated to 70 °C for 10 min and then 2-(bromomethyl)­naphthaline (19.00 g, 85.9 mmol, 2 equiv) was added. Heating was continued until TLC analysis showed full consumption of the starting material (16 h). Subsequently, ethyl acetate was added to the reaction and solids were filtered off. After concentration of the filtrate, the crude product was purified via column chromatography (250 g SiO_2_, 2:1 to 1:2 PE/EA) to yield 14.33 g (81%) of a white crystalline solid.

^**1**^ **H NMR** (400 MHz, CDCl_3_) δ 7.91–7.81 (m, 4H, Nap-*H*), 7.60–7.39 (m, 3H, Nap-*H*), 7.08–6.96 (m, 2H, Ph-*H*), 6.91–6.73 (m, 2H, Ph-*H*), 4.95 (d, *J* = 2.1 Hz, 2H, C*H*2), 4.70 (d, *J* = 7.8 Hz, 1H, *H-1*), 4.03 (dd, *J* = 9.4, 7.8 Hz, 1H, *H-2*), 3.83 (dd, *J* = 3.4, 1.2 Hz, 1H, *H-4*), 3.76 (s, 3H, O–CH3), 3.63 (qd, *J* = 6.5, 1.2 Hz, 1H, *H-5*), 3.55 (dd, *J* = 9.4, 3.3 Hz, 1H, *H-3*), 2.82–2.10 (b, 2H, OH), 1.37 (d, *J* = 6.5 Hz, 3H, *H*-6).

^**13**^ **C­{** ^**1**^ **H} NMR** (101 MHz, CDCl_3_) δ 155.4 (*Ph*-OMe), 151.2 (*Ph*–O-C-1), 135.1 (*Nap*), 133.2 (*Nap*), 128.6 (*Nap*), 127.9 (*Nap*), 127.8 (*Nap*), 126.9 (*Nap*), 126.2 (*Nap*), 118.8 (Ph), 114.5 (Ph), 102.4 (*C-1*), 80.4 (*C-3*), 72.3 (−*C*H_2_−), 70.8­(C-2), 70.6 (*C-5*), 69.1 (*C-4*), 55.6 (-O*Me*), 16.4 (*C-6*).

**HRMS (ESI)** *m*/*z*: [M + Na]^+^ Calcd for C_24_H_26_NaO_6_ 433.1621; found 433.1636.

#### (4-Methoxy)­phenyl 2,4-diazido-2,4-dideoxy-3-O-(2-naphthylmethyl)-β-L-rhamopyranoside (**6**)

A solution of compound **5** (10.3 g, 25.1 mmol, 1.0 equiv) in CH_2_Cl_2_ (200 mL) and pyridine (10.1 mL, 125.0 mmol, 5.0 equiv) was cooled to 0 °C with an ice bath and triflic anhydride (9.25 mL, 55.2 mmol, 2.2 equiv) was added dropwise over a 10 min period. After 1 h of stirring at 0 °C, TLC analysis showed full conversion of the starting material. Subsequently, the reaction was diluted with CH_2_Cl_2_, poured onto 1 M HCl and the phases were separated. The aqueous phase was then additionally extracted twice witch CH_2_Cl_2_ and the combined organic phases were washed with aq. sat. NaHCO_3_ then dried over MgSO4, filtered and finally concentrated *in vacuo*. The remaining viscous oil was then dissolved in toluene (120 mL) and tetrabutylammonium azide (15.00 g, 52.7 mmol, 2.1 equiv) was added and the mixture was heated to 80 °C until TLC analysis showed consumption of starting material (ca. 4 h). The solvent was evaporated from the reaction mixture and the resulting crude product was purified by flash chomratography, using 40 g of Celite for a solid application (250 g SiO2, 4:1 1:2 PE/EE) to yield 10.82 g (94%) of a white, amorphous solid.

^**1**^ **H NMR** (400 MHz, CDCl_3_) δ 7.93–7.83 (m, 4H, Nap-*H*), 7.61–7.56 (m, 1H, Nap-*H*), 7.54–7.47 (m, 2H, Nap-*H)*. 6.95–6.88 (m, 2H, Ph-*H*), 6.82–6.76 (m, 2H, Ph-*H*), 4.93 (dd, *J* = 16.2, 12.0 Hz, 2H, C*H*2), 4.86 (d, *J* = 1.2 Hz, 1H, *H-1*), 4.11 (dd, *J* = 3.4, 1.2 Hz, 1H, *H-2*), 3.76 (s, 3H, O–CH3), 3.59 (dd, *J* = 9.7, 3.4 Hz, 1H, *H-3*), 3.51 (dd, *J* = 9.7, 9.3 Hz, 1H, *H-4*), 3.21 (qd, *J* = 9.6, 6.1 Hz, 1H, *H-5*), 1.40 (d, *J* = 6.5 Hz, 3H, *H*-6) ppm.

^**13**^ **C­{** ^**1**^ **H} NMR** (101 MHz, CDCl_3_) δ 155.5 (*Ph*-OMe), 150.4 (*Ph*–O-C-1), 134.2 (*Nap*), 133.3 (*Nap*). 128.7 (*Nap*), 128.0 (*Nap*), 127.8 (*Nap*), 127.2 (*Nap*), 126.4 (*Nap*), 125.8 (*Nap*), 118.0 (Ph), 114.5 (Ph), 98.3 (*C-1*), 79.2 (*C-3*), 72.3 (−*C*H2−), 71.4 (*C-5*), 63.7 (*C-4*), 61.2 (*C-2)*, 55.6 (-O*Me*), 18.5 (*C-6*) ppm.

**HRMS (ESI)** *m*/*z*: [M + Na]^+^ Calcd for C_24_H_24_N_6_NaO_4_ 483.1751; found 483.1773.

### General Procedure for the Equatorially Selective Mono Azide Reduction

Diazido pyranoside (0.07 mmol, 1.0 equiv) was added to a round-bottom flask and dissolved in a 1:1 mix of methanol and pyridine (1.4 mL, 0.05 M). Subsequently, Lindlar catalyst was added (0.0035 mmol, 0.05 equiv) and the flask was evacuated and backfilled three times with hydrogen from a balloon. The reaction was then stirred under an atmosphere of hydrogen until TLC analysis revealed full conversion (2–16 h). Subsequently, the reaction was filtered, the solvent removed and the crude product was purified by flash chromatography to reveal the desired product.

#### (4-Methoxy)­phenyl 2-azido-4-amino-2,4,dideoxy-3-O-(2-naphthylmethyl)-β-L-rhamopyranoside (**7**)

Product synthesized according to the general procedure. Reduction under H_2_ atmosphere was accomplished after 16 h. Product obtained was a white amorphous solid.

Purification: 3 g SiO_2_, 100% EtOAc

Yield: 27 mg (87%).

^**1**^ **H NMR (**400 MHz, CDCl_3_) δ 7.89–7.1 (m, 4H, Nap-*H*), 7.56–7.46 (m, 3H, Nap-*H*), 6.95 (m, 2H, Ph-*H*), 6.81 (m, 2H, Ph-*H*), 4.94 (s, 1H, *H-1*), 4.93 (d, *J* = 10.9 Hz, 1H, −C*H*_2_−), 4.75 (d, *J* = 10.7 Hz, 1H, −C*H*_2_−), 4.16 (dd, *J* = 3.3, 1.3 Hz. One H, *H-2*), 3.77 (s, 3H, -*OMe*), 3.44 (dd, *J* = 9.9, 3.7 Hz, 1H, *H-3*), 3.34 (dddd, *J* = 9.3, 6.2 Hz, 1H, *H-5*), 2.98 (dd, *J* = 9.9, 9.3 Hz, 1H, *H-4*), 1.36 (d, *J* = 6.1, 3H, *H-6*) ppm.

^**13**^ **C­{** ^**1**^ **H} NMR** (101 MHz, CDCl_3_) δ 155.4 (*Ar*-OMe), 150.6 (*Ph*–O-C-1), 134.5 (*Nap*), 133.2 (*Nap*), 128.8 (*Nap*), 128.0 (*Nap*), 127.8 (*Nap*), 127.2 (*Nap*), 126.4 (*Nap*), 126.3 (*Nap*), 125.8 (*Nap*), 118.00 (*Ar*), 114.5 (*Ar*), 98.5 (*C-1*), 80.6 (*C-3*), 73.6 (*C-5*), 71.8 (−*C*H2−) 60.5 (*C-2*), 55.6 (*-OMe*), 53.5 (*C-4*), 18.1 (*C-*6) ppm.

**HRMS (ESI)** *m*/*z*: [M + H]^+^ Calcd for C_24_H_27_N_4_O_4_ 435.2027; found 435.2046.

#### (4-Methoxy)­phenyl 2-azido-4-(2′,2′,2′ trichloroethoxycarbonyl)-amino-2,4,dideoxy-3-O-(2-naphthylmethyl)-β-L-rhamopyranoside (**8**)

Diazido rhamnoside **6** (4.60 g, 10.0 mmol, 1.0 equiv) was dissolved in methanol (100 mL) and pyridine (100 mL) was added. Subsequently, Lindlar catalyst was added (1.1 g, 0.5 mmol, 0.05 equiv) and the flask was evacuated and backfilled with hydrogen from a balloon three times. The reaction was then stirred under an atmosphere of hydrogen for 3 h until TLC analysis revealed full conversion. Subsequently, the reaction was filtered through a plug of Celite and the solvent evaporated. The crude product was dissolved in dichloromethane (50 mL) and cooled to 0 °C with an ice bath. To this was added N,N diisopropylethylamine (2.6 mL, 15 mmol, 1.5 equiv) and finally TrocCl was added dropwise (1.8 mL, 13 mmol, 1.3 equiv). The reaction was allowed to warm to 25 °C over 16 h and then water was added to the reaction. The mixture was extracted three times with dichloromethane. The combined organic phases were dried over MgSO_4_, filtered and concentrated. The crude product was purified by flash chromatography (90 g SiO2, 2:1 PE/EE) to yield 5.01 g (82%) of a white crystalline solid.

^**1**^ **H NMR** (400 MHz, CDCl_3_) δ 7.91–7.77 (m, 4H, Nap-*H*), 7.54–7.48 (m, 1H, Nap-*H*), 7.54–7.47 (m, 2H, Nap-*H)*. 6.97–6.91 (m, 2H, Ph-*H*), 6.83–6.77 (m, 2H, Ph-*H*), 4.97 (b, 1H, *H-1*), 4.91 (d, *J* = 11.7 Hz, 1H, −C*H*_2_−), 4.72 (d, *J* = 12.1 Hz, 1H −C*H*_2_−), 4.67 (d, *J* = 12.1 Hz, 1H, −C*H*_2_CCl_3_), 4.56 (d, *J* = 12.1 Hz, 1H, −C*H*_2_CCl_3_), 4.22 (d, *J* = 2.7 Hz, 1H, *H-2*), 3.93 (dd, J= 10.2, 3.5 Hz, 1H, *H-3*) 3.79–3.71 (m, 1H, *H-5*), 3.76 (s, 3H, O–CH_3_), 3.53–3.44 (m, 1H, *H-4*), 1.34 (d, *J* = 6.2 Hz, 3H, *H*-6) ppm.

^**13**^ **C­{** ^**1**^ **H} NMR** (101 MHz, CDCl_3_) δ 155.4 (*Ph*-OMe), 154.0 (−NH_2_ *C*(O)­CH_2_−), 150.6 (*Ph*–O-C-1), 134.5 (*Nap*), 133.2 (*Nap*), 128.7 (*Nap*), 128.0 (*Nap*), 127.8 (*Nap*), 127.2 (*Nap*), 126.4 (*Nap*), 125.8 (*Nap*), 118.0 (Ph), 114.5 (Ph), 98.2 (*C-1*), 95.5 (−CH_2_ *C*Cl_3_), 75.5 (*C-3*), 74.3 (−*C*H_2_CCl_3_), 71.8 (−*C*H_2_−), 70.6 (C-5), 61.5 (*C-2*), 55.6 (-O*Me*), 55.1 (*C-4*), 18.0 (*C-6*) ppm.

**HRMS (ESI)** *m*/*z*: [M + H]^+^ Calcd for C_27_H_28_Cl_3_N_4_O_6_^+^ 609.1069; found 609.1083.

## Supplementary Material



## Data Availability

The data underlying this study are available in the published article and its Supporting Information.

## Notes

Synthesis of compounds **6a–6e** and products **7a–c**, as well as ^1^H, ^13^C NMR spectra and HRMS data for all new compounds. Additionally, comparison of HSQC spectra of compounds **6** and **7** (PDF)
