# Supplementary material for: Selective Monoreduction of 2,4 Diazido-Dideoxy Hexoses by Hydrogenation over Lindlar Catalyst
Source: J Org Chem. 2025 Dec 2;90(49):17588–93. doi: 10.1021/acs.joc.5c02433 (PMC12706787; doi:10.1021/acs.joc.5c02433)

Supporting information

Selective mono-reduction of 2,4 diazido-dideoxy hexoses by hydrogenation over Lindlar Catalyst

Leonie Wiener, Philipp J. Gritsch*, Maximilian Kaiser, Nicolas Kratena, Peter Gärtner

Institute of Applied Synthetic Chemistry, TU Wien, A-1060 Wien

Table of Contents

1 MATERIALS AND METHODS: CHEMICALS AND SOLVENTS....S3

2 EXPERIMENTAL PROCEDURES: SYNTHESIS OF STARTING MATERIALS....S4

3 EQUATORIALLY SELECTIVE MONO AZIDE REDUCTION....S14

4 HSQC SPECTRA OF COMPOUNDS 6 AND 7....S17

5 REFERENCES....S18

6 ^{1}H AND ^{13}C SPECTRA....S19

1 Materials and Methods: Chemicals and solvents

1.1 Chemicals and solvents

Unless stated otherwise, all chemicals were purchased from commercial suppliers (Sigma-Aldrich,BLDPharm, TCI, abcr, Acros, Fisher, VWR, FluoroChem, Angene) and used without further purification. Lindlar catalyst was purchased from TCI, Product. No 1703: Palladium 5% on Calcium Carbonate (poisoned with Lead). Some dry solvents (toluene, CH_2Cl_2, THF, Et_2O) were obtained from a PureSolv SPS system by Innovative Technologies. Dry MeCN, pyridine, and all other dry solvents were obtained from Acros Organics over molecular sieves and used without further purification. All other solvents used were HPLC grade or p.a. unless stated otherwise. Unless otherwise, reactions were carried out at 25 °C. Reactions that require heating were heated to the described temperature with an oilbath with a temperature probe inserted into the oil for temperature control.

Caution: Organic azides, especially multiple azido compounds, are potentially hazardous and explosive. Although there have been no serious incidents in our study, all manipulations should be done carefully in a hood with a glass shield to avoid detonation. Sodium azide as well as tetrabutylammonium azide should be handled with a plastic spatula.

1.2 Glassware and reaction conditions

Reactions were carried out in round bottom flasks, oven-dried Schlenk flasks or microwave vials under an inert atmosphere (Argon) unless stated otherwise.

1.3 Analytical techniques

^{1}H, and ^{13}C NMR spectra were recorded on a Bruker AVIII 400 Spectrometer (^{1}H: 400 MHz and ^{13}C: 101 MHz) or a Bruker Avance III 600 (^{1}H: 600 MHz and ^{13}C: 151 MHz) in, $CDCl_3$ or CD_3OD and referenced to residual solvent peaks. Chemical shifts δ are quoted in parts per million (ppm) to the nearest 0.01 for ^{1}H and 0.1 for ^{13}C, coupling constants J are quoted in Hz to the nearest 0.1 and splitting are recorded as singlet (s), doublet (d), triplet (t), doublets of doublets (dd), doublets of doublets of doublets (ddd), doublets of triples (dt). Assignments were based upon COSY, HSQC and HMBC experiments. Any grease or residual solvent impurity will be indicated in the spectrum.

1.4 Chromatography

Analytical thin layer chromatography was performed on pre-coated silica gel aluminium sheets from Merck (TLC Silica Gel 60 F254). Spots were visualized either by the quenching of UV fluorescence or by staining with phosphomolybdic acid/cerium sulfate, or acidic p-anisaldehyde or vanillin solutions. Preparative column chromatography was carried out using Geduran Silica Gel 60 (40 – 63 µm) from Merck or LiChroprep® RP-18 (25 – 40 µm), which

will be indicated as “fine silica”. In cases where mixtures of solvents were used, the ratios refer to the component volumes. In cases where gradients where used, the start and the end ratio are stated.

1.5 High-resolution mass spectrometry

The HR-MS analysis was carried out from methanol or acetonitrile or water or a mixture of these solvents (concentration: 10 µM) by using an Agilent G7167B multi sampler, an Agilent G7120A binary pump with degasser, an Agilent G7116B oven and Agilent 6545 Q-TOF mass spectrometer equipped with a dual AJS ion score.

2 Experimental procedures: synthesis of starting materials

2.1 Synthetic routes overview

Synthetic route for **6a**

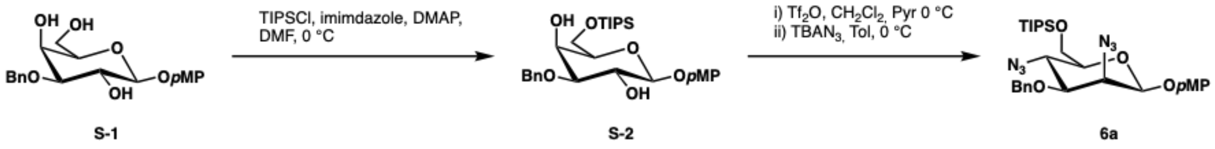

Synthetic route for **6b**

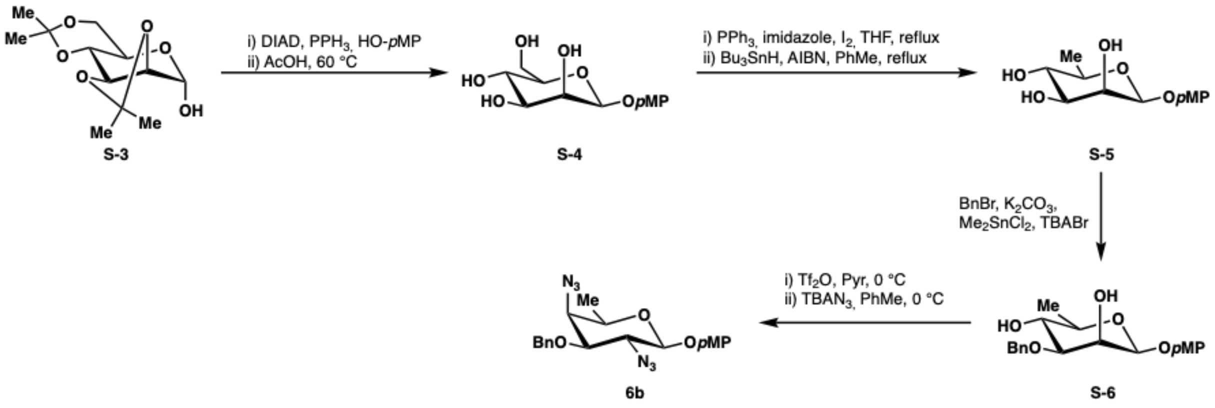

Synthetic route for **6c**

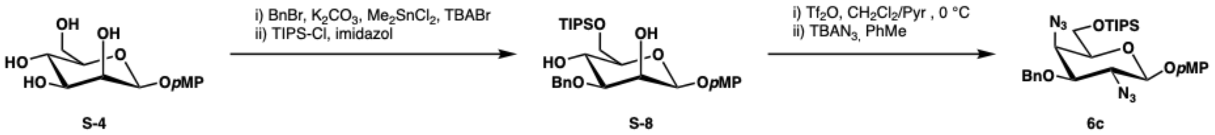

Synthetic route for **6d**

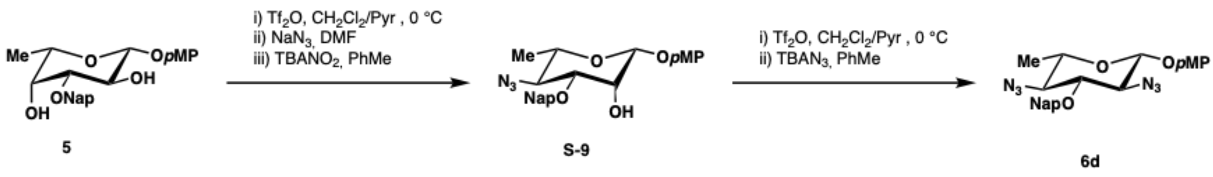

Synthetic route for **6e**

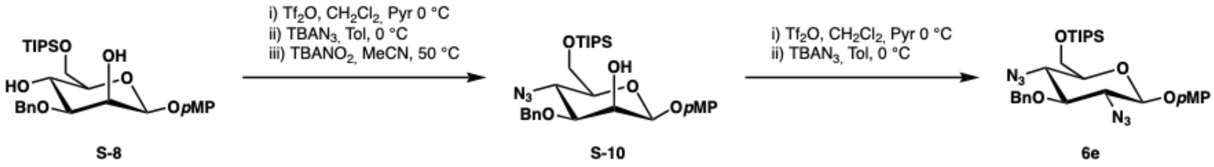

2.2 (4-Methoxy)phenyl 3-O-benzyl-6-O-triisopropylsilyl-β-D-galactopyranoside (S-2)

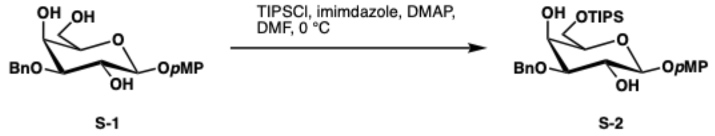

A flask was charged with (4-methoxy)phenyl 3-O-benzyl-β-D-galactopyranoside[2] (**S-1**) (321 mg, 0.853 mmol, 1.0 eq.), imidazole (139 mg, 2.05 mmol 2.4 eq.) and DMAP (11 mg, 0.09 mmol, 0.1 eq.). This mixture was dissolved in DMF (0.9 mL) and cooled to 0°C. Then TIPSCl (0.23 mL, 1.02 mmol, 1.2 eq.) was added and the reaction then was stirred for 5 h after which the solvent was evaporated under reduced pressure. The crude product was purified using column chromatography (50 g, 1:2 PE:EE) obtaining product as a white amorphous solid (445 mg, 98 %).

^{1}H NMR (400 MHz, $CDCl_3$) δ 7.44 – 7.28 (m, 5H, *H*Ar), 7.03 (m, 2H, *H*Ar), 6.80 (m, 2H, *H*Ar), 4.79 (s, 2H, CH_2), 4.73 (d, *J* = 7.8 Hz, 1H, *H-1*), 4.10 (dd, *J* = 3.3, 1.1 Hz, 1H, *H-4*), 4.06 (dd, *J* = 9.5, 7.8 Hz, 1H, *H-2*), 4.01 (dd, *J* = 10.1, 6.0 Hz, 1H, *H-6a*), 3.93 (dd, *J* = 10.1, 5.7 Hz, 1H, *H-6b*), 3.77 (s, 3H, CH_3O), 3.53 (td, *J* = 5.9, 1.1 Hz, 1H, *H-5*), 3.50 (dd, *J* = 9.4, 3.3 Hz, 1H, *H-3*), 1.19 – 0.98 (m, 21H, *TIPS*) ppm.

^{13}C{^{1}H} NMR (101 MHz, $CDCl_3$) δ 155.4 (*Ar*-OMe), 151.3 (*Ar*-O-C-1) 137.8 (*Ar*-CH_2), 128.7 (2C , *Ar*), 128.1 (*Ar*), 127.9 (2C, *Ar*), 118.7 (2C, *Ar*), 114.4 (*Ar*), 102.5 (*C-1*), 80.5 (*C-3*), 75.2 (*C-5*), 72.2 (*CH_2*), 70.9 (*C-2*), 66.2 (*C-4*), 62.6 (*C-6*), 55.6 (CH_3O), 17.9 (*TIPS*), 17.9 (*TIPS*), 11.9 (*TIPS*) ppm.

HRMS (ESI) m/z: $[M+H]^+$ Calcd. for $C_{29}H_{45}O_7Si$ 533.2929; found 533.2928.

2.3 (4-Methoxy)phenyl 2,4-azido-2,4-deoxy-3-O-benzyl-6-O-triisopropylsilyl-β-D-mannopyranoside (6a)

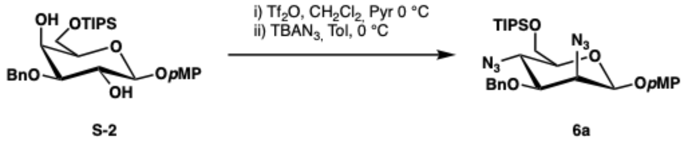

Compound **S-2** (2220 mg, 4.17 mmol. 1.0 eq.) was added to a flask and dissolved in CH_2Cl_2 (20.0 mL) and pyridine (1.0 mL). The solution was cooled to 0 °C using an ice bath and a Tf_2O (1 M in CH_2Cl_2, 10.4 mL, 10.4 mmol, 2.5 eq.) was added dropwise. The mixture was stirred for 2.5 h at 0 °C and then for 2 h at 25 °C. Subsequently it was diluted CH_2Cl_2 and poured onto 1 N HCl. The aqueous phase was extracted three times with CH_2Cl_2 and the combined organic phases were washed with saturated $NaHCO_3$ solution. The organic phase was then dried over $MgSO_4$, filtered and the solvent was evaporated. The crude product was used without further purification. The intermediate triflated product was dissolved in toluene (8 mL), $TBAN_3$ (2.90 g, 9.69 mmol, 2.3 eq.) was added, and the solution was stirred at 70 °C for 4 h. The solvent was evaporated and the crude product was purified using column chromatography (80 g, 20:1 to 4:1 PE/EtOAc) to yield the desired diazide (2.18 g, 93% over two 2 steps) as a colorless crystalline solid.

^{1}H NMR (400 MHz, $CDCl_3$) δ 7.46 – 7.31 (m, 5H, *H*Ar), 6.98 (m, 2H, *H*Ar), 6.77 (m, 2H, *H*Ar), 4.86 (d, J = 1.4 Hz, 1H, *H-1*), 4.78 (d, J = 11.8 Hz, 1H, C*H*$_2$) 4.74 (d, *J* = 11.4 Hz, 1H, C*H*$_2$), 4.08 (dd, J = 3.6, 1.0 Hz, 1H, *H-2*), 3.98 (dd, J = 11.0, 1.8 Hz, 1H, *H-6a*), 3.90 (dd, J = 11.2 Hz, 5.2 Hz, 1H, *H-6b*), 3.82 (dd, J = 9.9Hz, 1H, *H-4*) 3.76 (s, 3H, O*Me*), 3.57 (dd, J = 9.7, 3.5 Hz, 1H, *H-3*) 3.17 (ddd, J = 10.1, 5.3, 1.8 Hz, 1H, *H-5*) 1.12-1.02 (m, 21H, *TIPS*) ppm.

^{13}C{^{1}H} NMR (101 MHz, $CDCl_3$) δ 155.5 (*Ar*-OMe), 150.7 (*Ar*-O-C-1), 136.8 (*Ar*-CH_2), 128.7 (*Ar*), 128.4 (*Ar*), 128.2 (*Ar*), 118.4 (*Ar*), 114.4 (*Ar*), 98.9 (*C-1*), 79.5 (*C-3*), 76.4 (*C-5*), 72.2 (*C*H$_2$), 63.1 (*C-6*), 60.9 (*C-2*), 58.0 (*C-4*), 55.6 (O*Me*), 17.9 (TIPS), 17.9 (TIPS), 11.9 (TIPS) ppm.

HRMS (ESI) m/z: $[M+Na]^+$ Calcd. for $C_{29}H_{42}N_6NaO_5Si$ 605.2878; found 605.2892.

2.4 (4-Methoxy)phenyl β-D-mannopyranoside (S-4)

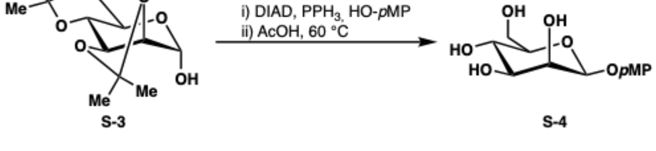

2,3:4,6-Di-O-isopropylidene α-Mannose (**S-3**)[3] (2480 mg, 9.5 mmol, 1.0 eq.) was dissolved in toluene (70 mL) and to this was added *p*-Methoxyphenol (1774 mg, 14.3 mmol, 1.5 eq)

triphenylphosphine (3787 mg, 14.3 mmol. 1.5 eq) and finally DIAD (2.81 ml, 14.3 mmol, 1.5 eq.) was added. The resulting orange solution was stirred at room temperature for 16 h and then water was added. The resulting mixture was extracted three times with diethyl ether and the combined organic phases were subsequently washed with brine, dried over MgSO4 and filtered. Purification by flash chromatography (400 g Sio3, 10:1 – 1:1 PE/EtOAc) gave the desired β-mannopyranoside which was dissolved in 90% acetic acid (10 mL) and heated to 64 °C for 2 h. Then the reaction was concentrated *in vacuo* and the resulting crude product was purified by column chromatography (40 g SiO2, 1-20% MeOH in CH_2Cl_2) to reveal 546 mg (20% over two steps) of the desired product as a colorless crystalline solid.

^{1}H NMR (400 MHz, d4-methanol) δ 7.05 (m, 2H, *Ph*), 6.85 (m, 2H, *Ph*). 5.08 (d, J = 1.0 Hz, 1H, *H-1*), 4.06 (dd, J = 3.0, 0.9 Hz, 1H *H-2*), 3.91 (dd, J = 12.5, 2.4 Hz, 1H, *H-6a*), 3.76 (dd, J = 11.8 Hz, 6.0 Hz, 1H, *H-6b*), 3.76 (s, 3H, -O*Me),* 3.67 (dd, J = 9.5, 9.5 Hz, 1H, *H-4*), 3.57 (dd, J = 9.4, 3.2 Hz, 1H, *H-3*) 3.40-3.34 (m, 1H, *H*-5) ppm.

^{13}C{^{1}H} NMR (101 MHz, d4-methanol) δ 155.1 (*Ph*-OMe), 151.3 (*Ph*-O-C-1), 117.4 (2C, Ph), 114.1 (2C, Ph), 99.0 (*C-1*), 77.0 (*C-5*), 73.8 (*C-3*), 71.2 (*C-2*), 67.0 (*C-4*), 61.3 (*C-6*), 54.7 (O*Me)* ppm.

HRMS (ESI) m/z: $[M+Na]^+$ Calcd. for $C_{13}H_{18}NaO_7$ 309.0945; found 309.0947.

2.5 (4-Methoxy)phenyl β-D-rhamnopyranoside (S-5)

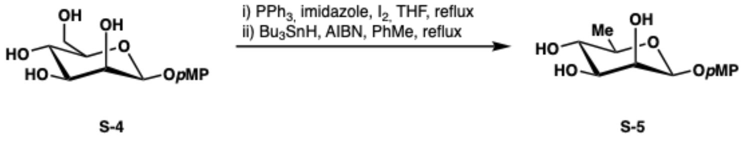

4-Methoxyphenyl-β-D-mannopyranoside (**S-4**) (400 mg, 1.40 mmol, 1.0 eq.) was dissolved in dry THF (14 mL) under argon. Triphenylphosphine (555 mg, 2.1 mmol, 1.5 eq.) and imidazole (192 mg, 2.79 mmol, 2.0 eq.) were added, and the reaction mixture was heated to reflux for 1 h. After cooling of the solution to room temperature, iodine (532 mg, 2.1 mmol, 1.5 eq.) was added, and the reaction mixture was again heated to reflux and stirred for 2 h. The cooled reaction mixture was then poured onto a stirred mixture of ice, aq. sat. $NaHCO_3$ solution and ethyl acetate. Subsequently the phases were separated, and the aqueous layer was extracted with ethyl acetate three times. The combined organic phases were dried over $MgSO_4$, and the solvent was evaporated via rotary evaporation. The crude product was purified by eluting with ethylacetate through a plug of SiO_2 and subsequently added to a flask and dissolved in toluene (13 mL). Bu_3SnH (545 mg, 1.82 mmol, 1.3 eq.) and AIBN (23 mg, 0.14 mmol, 0.1 eq.) were added, and the reaction mixture was refluxed and stirred for 4 h and subsequently cooled to room temperature. The solvent was evaporated and the crude product was purified via column

chromatography (80 g, 1:4 to 0:1 PE/EE) to obtain the desired product (255 mg, 68 %) as a white crystalline solid.

^{1}H NMR (400 MHz, d4-methanol) δ 6.99 (m, 2H, *Ph*), 6.83 (m, 2H, *Ph*), 5.02 (d, *J* = 1.1 Hz, 1H, *H-1*), 4.04 (dd, *J* = 3.2, 1.1 Hz, 1H, *H-2*), 3.74 (s, 3H, *CH_3O*), 3.50 (dd, *J* = 9.2, 3.3 Hz, 1H, *H-3*), 3.43 – 3.33 (m, 2H, *H-4, H-5*), 1.33 (d, *J* = 5.9 Hz, 3H, C*H_3*) ppm.

^{13}C{^{1}H} NMR (101 MHz, d4-methanol) δ 155.1 (*Ph*-OMe), 151.3 (*Ph*-O-C-1), 117.4 (*Ar*), 114.1 (*Ar*), 98.9 (*C-1*), 73.5 (*C-3*), 72.3 (*C-4*), 72.3 (*C-5*), 71.3 (*C-2*), 54.7 (*C*H_3O), 16.6 (*C-6*) ppm.

HRMS (ESI) m/z: $[M+Na]^+$ Calcd. for $C_{13}H_{18}NaO_6$ 293.0995; found 293.0998.

2.6 (4-Methoxy)phenyl 3-O-benzyl-β-D-rhamnopyranoside (S-6)

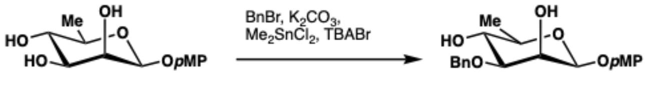

S-5 S-6

Compound **S-5** (200 mg, 0.74 mmol, 1.0 eq.), TBABr (24 mg, 0.08 mmol, 0.1 eq.), K_2CO_3 (155 mg, 1.11 mmol, 1.5 eq.) and dimethyltin dichloride (16 mg, 0.08 mmol, 0.1 eq.) were dissolved in acetonitrile (3.5 mL) and DMF (0.35 mL) and stirred for 10 min. To this solution was added benzyl bromide (0.18 mL, 1.48 mmol. 2.0 eq.) and the mixture was heated to 70 °C for 5. Then, the solids were removed through filtration and the solvent was evaporated. The crude product was purified via column chromatography (30 g, 1:1 PE:EA) to give **S-6** as a coloroless solid (146 mg, 55 %).

^{1}H NMR (400 MHz, $CDCl_3$) δ 7.45 – 7.28 (m, 5H, *H*Ar), 6.99 (d, *J* = 9.2 Hz, 2H, *H*Ar), 6.82 (d, *J* = 9.2 Hz, 2H, *H*Ar), 4.93 (d, *J* = 1.2 Hz, 1H, *H-1*), 4.83 (d, *J* = 11.7 Hz, 1H, -C*H_2*-), 4.60 (d, *J* = 11.8 Hz, 1H, C*H_2*), 4.32 (d, *J* = 3.0 Hz, 1H, *H-2*), 3.77 (s, 3H, C*H_3*O), 3.70 (dd, *J* = 9.2, 9.2 Hz, 1H, *H-4*), 3.46 – 3.35 (m, 2H, *H-3*, *H-5*), 2.47 (s, 1H, O*H*), 2.25 (s, 1H, O*H*), 1.40 (d, *J* = 6.1 Hz, 3H, C*H_3*) ppm.

^{13}C{^{1}H} NMR (101 MHz, $CDCl_3$) δ 155.3 (*Ph*-OMe), 150.9 (*Ph*-O-C-1), 137.4 (*Ar*), 128.8 (*Ar*), 128.3 (*Ar*), 128.1 (*Ar*), 118.8 (*Ar*), 114.5 (*Ar*), 99.0 (*C-1*), 80.9 (*C-3*), 72.0 (*C-5*), 71.2 (*C-4*), 72.1 (*C*H_2), 68.0 (*C-2*), 55.6 (*C*H_3O), 17.8 (*C-6*) ppm.

HRMS (ESI) m/z: $[M+K]^+$ Calcd. for $C_{20}H_{24}KO_6$ 399.1204; found 399.1211.

2.7 (4-Methoxy)phenyl 2,4-diazido-2,4-deoxy-3-O-benzyl-β-D-fucopyranoside (6b)

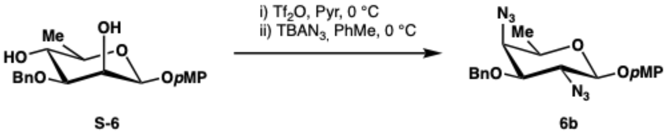

Compound **S-6** (75 mg, 0.21 mmol, 1.0 eq.) was added to a flask and dissolved in CH_2Cl_2 (1.2 mL) and pyridine (0.4 mL). The solution was cooled to 0 °C using an ice bath and a 1 M solution of Tf_2O in CH_2Cl_2 (0.62 mL, 0.62 mmol, 3.0 eq.) was added dropwise. The mixture was stirred for 3 h and poured onto a saturated $NaHCO_3$ solution. The aqueous phase was extracted three times with CH_2Cl_2 and the combined organic phases were washed with 1 N HCl. The organic phase was dried over $MgSO_4$, filtered and the solvent was evaporated. The residue was dissolved in toluene (1 mL) and the solution was cooled to 0 °C using an ice bath. $TBAN_3$ (187 mg, 0.62 mmol, 3.0 eq.) was added, and the solution was stirred for 18 h. Subsequently, the solvent was evaporated and the crude product was purified using column chromatography (50 g, 5:1 to 3:1 PE:EA) to yield compound **6b** (58 mg, 67 %) as a colorless amorphous solid.

^{1}H NMR (600 MHz, $CDCl_3$) δ 7.45 – 7.31 (m, 5H, *H*Ar), 7.01 (d, *J* = 9.1 Hz, 2H, *H*Ar), 6.81 (d, *J* = 9.2 Hz, 2H, *H*Ar), 4.77 (d, *J* = 2.1 Hz, 2H, C*H₂*), 4.60 (d, *J* = 8.1 Hz, 1H, *H-1*), 3.90 (dd, *J* = 10.3, 8.1 Hz, 1H, *H-2*), 3.77 (s, 3H, C*H₃*O), 3.65 (dd, *J* = 3.7, 1.5 Hz, 1H, *H-4*), 3.59 (dd, *J* = 6.5, 1.3 Hz, 1H, *H-5*), 3.50 (dd, *J* = 10.1, 3.7 Hz, 1H, *H-3*), 1.35 (d, *J* = 6.3 Hz, 3H, C*H₃*) ppm.

^{13}C{^{1}H} NMR (151 MHz, $CDCl_3$) δ 155.6 (*Ph*-OMe), 151.1 (*Ph*-O-C-1), 137.0 (*Ar*), 128.7 (*Ar*), 128.3 (*Ar*), 128.1 (*Ar*), 118.8 (*Ar*), 114.5 (*Ar*), 101.8 (*C-1*), 79.5 (*C-3*), 72.8 (*C*H₂), 69.4 (*C-5*), 62.5 (*C-2*), 62.4 (*C-4*), 55.6 (*C*H₃O), 17.6 (*C-6*) ppm.

HRMS (ESI) m/z: $[M+Na]^+$ Calcd. for $C_{20}H_{22}N_6NaO_4$ 433.1595; found 433.1614.

2.8 (4-Methoxy)phenyl 3-*O*-benzyl-β-D-mannopyranoside (S-8)

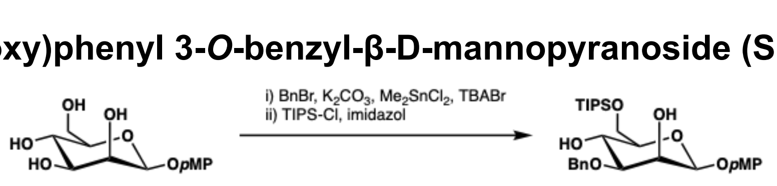

Compound **S-4** (830 mg, 2.90 mmol, 1.0 eq.), TBABr (96 mg, 0.29 mmol, 0.1 eq.), K_2CO_3 (607 mg, 4.35 mmol. 1.5 eq.) and dimethyltin dichloride (64 mg, 0.29 mmol, 0.1 eq.) were dissolved in a 10:1 mixture of acetonitrile (20 mL) and DMF (2 mL) and stirred for 10 min. To this solution benzyl bromide (0.70 mL, 5.80 mmol, 2.0 eq.) was added and the mixture was heated to 70 °C

for 5 . Then the solids were removed through filtration and the solvent was evaporated. The crude product was purified via column chromatography (80 g, 0-15% MeOH in CH_2Cl_2) to yield an intermediate product (573 mg). This intermediate product (573 mg, 1.52 mmol) was dissolved in DMF (1.5 mL) and imidazole (228 mg, 3.35 mmol, 2.2 eq.) as well as TIPSCl (323 mg, 1.67 mmol, 1.1 eq.) were added. This mixture was stirred at 25 °C until TLC analysis showed consumption of the intermediate product, upon which the reaction was poured onto water and extracted three times with ethyl acetate. Combined organic phases were washed with brine, dried over MgSO4, filtered and concentrated. Purification by chromatography (90 g SiO2, 4:1 to 3:1 PE/EE) gave the desired product (612mg, 40% over two steps) as a white crystalline solid.

^{1}H NMR (600 MHz, $CDCl_3$) δ 7.42 (m, 2H, Ph*H*), 7.37 (m, 2H, Ph*H*) 7.32 (m, 1H, Ph*H)*, 7.00 (m. 2H, Ph*H),* 6.80 (m, 2H, Ph*H)* 4.92 (d, *J* = 0.8 Hz, 1H, *H-1*), 4.82 (d, J = 12.0 Hz, 1H, -C*H₂*-), 4.76 (d, *J* = 12.3 Hz, 1H, -C*H₂-*), 4.26 (dd, *J* = 2.1, 2.1 Hz, 1H, *H-2*), 4.05-3.97 (m, 3H, *H-6a,b*, *H-4*), 3.77 (s, 3H, C**H₃**O), 3.48 (dd, *J* = 9.0, 3.1 Hz, 1H, *H-3*), 3.44 (ddd, *J* = 9.4, 5.7, 5.7 Hz, 1H, *H-5*), 3.15 (s, 1H, -*OH*), 2.51 (d, J= 2.5 Hz, 1H, -*OH*), 1.05 (b, 21H, *TIPS*) ppm.

^{13}C{^{1}H} NMR (151 MHz, $CDCl_3$) δ 155.3 (*Ar*-OMe), 151.0 (*Ar*-O-C-1) 137.7 (*Ar*-CH_2) 128.7 (*Ar,* 2C), 128.1 (*Ar*), 128.1 (*Ar***,** 2C), 118.1 (*Ar*, 2C), 114.4 (*Ar*, 2C), 99.1 (*C-1*), 80.7 (*C-3*), 75.0 (*C-5*), 71.7 (CH_2), 69.3 (*C-4*), 68.2 (*C-2*), 65.1 (*C-6*), 55.6 (-OCH_3), 17.9 (*TIPS*), 11.97 (*TIPS*) ppm.

HRMS (ESI) m/z: $[M+Na]^+$ Calcd. for $C_{29}H_{44}NaO_7Si$ 555.2748; found 555.2751.

2.9 (4-Methoxy)phenyl 2,4-diazido-2,4-deoxy-3-O-benzyl-β-D-galactopyranoside (6c)

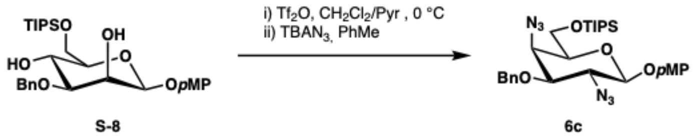

Compound **S-8** (200 mg, 0.38 mmol, 1.0 eq.) was added to a flask and dissolved in CH_2Cl_2 (2.1 mL) and pyridine (0.7 mL). The solution was cooled to 0 °C using an ice bath and Tf_2O (1M in CH_2Cl_2, 1.65 mL, 1.14 mmol, 3.0 eq.) was added dropwise. The mixture was stirred for 2 h and poured onto 1 N HCl. The aqueous phase was extracted three times with CH_2Cl_2 and the combined organic phases were washed with saturated $NaHCO_3$ solution. The organic phase was then dried over $MgSO_4$, filtered and the solvent was evaporated. The crude product was used without further purification. The intermediate triflated product was dissolved in toluene (2.1 mL) and the mixture was cooled to 0 °C using an ice bath. $TBAN_3$ (344 mg, 1.14 mmol, 3.0 eq.) was added, and the solution was removed from the ice beath and was stirred

for 18 h while warming to 25 °C. The solvent was evaporated and the crude product was purified using column chromatography (20 g, 15:1 PE:EA) to yield the desired diazide **6c** (204 mg, 92 % over 2 steps) as a colorless amorphous solid.

^{1}H NMR (600 MHz, $CDCl_3$) δ 7.45 – 7.31 (m, 5H, *H*Ar), 7.00 (m, 2H, *H*Ar), 6.80 (m, 2H, *H*Ar), 4.81 (d, *J* = 11.9 Hz, 1H, C*H2*), 4.76 (d, *J* = 11.7 Hz, 1H, C*H2*), 4.62 (d, *J* = 8.1 Hz, 1H, *H-1*), 3.95 (dd, *J* = 3.4, 1.1 Hz, 1H, *H-4*), 3.92 (dd, *J* = 10.1, 8.1, 1H, *H-2*), 3.89 – 3.78 (m, 2H, *H-6a*,*b*), 3.77 (s, 3H, C*H3*O), 3.52 (dd, *J* = 10.1, 3.5 Hz, 1H, *H-3*), 3.49 (ddd, *J* = 8.1, 5.7, 1.3 Hz, 1H, *H-5*), 1.05 (dd, *J* = 6.9, 3.3 Hz, 21H, *TIPS*) ppm.

^{13}C{^{1}H} NMR (151 MHz, $CDCl_3$) δ 155.6 (*Ar*-OMe), 151.0 (*Ar*-O-C-1), 137.0 (*Ar*-CH_2), 128.7 (*Ar*), 128.4 (*Ar*), 128.2 (*Ar*), 118.8 (*Ar*), 114.5 (*Ar*), 101.9 (*C-1*), 79.3 (*C-3*), 73.5 (*C-5*), 73.0 (*C*H_2), 62.9(*C-2*), 61.8 (*C-6*), 58.6 (*C-4*), 55.6 (*C*H_3O), 17.9 (*TIPS*), 11.8 (*TIPS*) ppm.

HRMS (ESI) m/z: $[M+Na]^+$ Calcd. for $C_{29}H_{42}N_6NaO_5Si$ 605.2878; found 605.2877.

2.10 (4-Methoxy)phenyl 4-azido-4-deoxy-3-O-(2-naphthylmethyl)-β-L-rhamopyranoside (S-9)

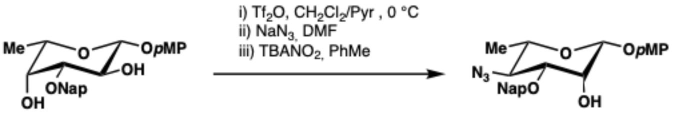

5

S-9

Compound **5** (616 mg, 1.53 mmol, 1.0 eq.) was dissolved in CH_2Cl_2 (6 mL) and pyridine (0.6 mL). The solution was cooled to 0 °C using an ice bath, and Tf_2O (1 M in CH_2Cl_2, 3.83 mL, 3.83 mmol, 2.5 eq.) was added dropwise. The mixture was stirred for 15 min at 0 °C and then for 30 min at 25 °C at which point TLC analysis showed full conversion of the starting material. At this point the reaction was poured onto 1 N HCl and the aqueous phase was extracted three times with CH_2Cl_2. The combined organic phases were then washed with aq. sat. $NaHCO_3$ and was dried over $MgSO_4$, filtrated and concentrated to yield the ditriflated intermediate product. This was then dissolved in DMF (4.0 mL) and cooled to 15 °C. NaN_3 (100 mg, 1.53 mmol, 1.0 eq.) was added, and the reaction was stirred for 4 h at which point TLC analysis showed full conversion. Subsequently the reaction was diluted with water and then extracted three times with diethyl ether. Combined organic phases were washed with brine, dried over $MgSO_4$, filtered and concentrated to yield a colorless oil which was dissolved in MeCN (3 mL) and $TBANO_2$ (531 mg,1.84 mmol, 1.2 eq.) was added. The mixture was then heated to 45 °C for 4 h and subsequently the solvent was distilled off. Purification (90 g SiO2, 4:1 to 3:2 PE/EtOAc) gave the desired compound (313 mg, 47% over 3 steps) as a lightly yellow solid.

^{1}H NMR (400 MHz, $CDCl_3$) δ 7.90-7.81 (m, 4H, Nap*H*), 7.58 (m, 1H, Nap*H*), 7.52–7.46 (m, 2H, Nap*H*), 6.95 (m, 2H, Ph*H*), 6.80 (m, 2H, Ph*H*), 4.97 (d, J = 12.0 Hz, 1H, C*H2*), 4.88 (d, J

= 12.0 Hz, 1H, C*H*$_2$), 4.82 (d, J = 1.0 Hz, 1H, *H-1*), 4.30 (dd, J = 2.9, 1.0 Hz, 1H, *H-2*), 3.76 (s, 3H, -C*H*$_3$), 3.60 (dd, J = 9.6, 9.6 Hz, 1H, *H-4*), 3.52 (dd, J = 9.6, 3.0, 1H, *H-3*), 3.24 (dq, J = 9.7, 6.1 Hz, 1H, *H-5*), 1.41 (d, J = 6.1 Hz, 3H, *H-6*) ppm.

^{13}C{^{1}H} NMR (101 MHz, CDCl$_3$) δ 155.4 (*Ar*-OMe), 150.7 (*Ar*-O-C-1) 134.5 (*Nap*-CH$_2$),133.2 (*Nap*), 128.6 (*Nap*), 128.0 (*Nap*), 127.8 (*Nap*), 127.1 (*Nap*), 126.3 (*Nap*), 126.2 (*Nap*), 125.8 (*Nap*), 118.1 (2C, Ar). 114.5 (2C, Ar), 98.7 (*C-1*), 79.6 (*C-3*), 71.5 (CH$_2$), 71.0 (*C-5*), 67.6 (*C-2*), 63.4 (*C-4*), 55.6 (CH$_3$O), 18.6 (*C-6*).

HRMS (ESI): m/z: [M+Na]$^+$ Calcd. for $C_{24}H_{25}N_3NaO_5$ 458.1686; found 458.1700.

2.11 (4-Methoxy)phenyl 2,4-diazido-2,4dideoxy-3-*O*-(2-naphthylmethyl)-β-L-quinovopyranoside (6d)

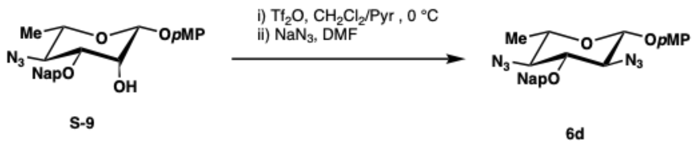

Compound **S-9** (220 mg, 0.51mmol, 1 eq.) was dissolved in CH$_2$Cl$_2$ (2 ml) and Pyridine (0.3 mL) and the mixture was cooled to 0 °C using an ice bath. To this was added Tf$_2$O (1 M in CH$_2$Cl$_2$, 0.61 mL, 0.61 mmol, 1.2 eq.) and the reaction was stirred at 0 °C for 1 h at which point TLC analysis showed complete conversion of the starting material. The reaction mixture was then poured on to 1N HCl and this mixture was then extracted three times with CH$_2$Cl$_2$. Combined organic phases were dried over MgSO$_4$, filtered and concentrated *in vacuo* to reveal the crude triflate intermediate product which was directly dissolved in DMF (1.5 mL) and sodium azide (40 mg, 0.61 mmol, 1.2 eq.) was added. This solution was then stirred at 25 °C for 3h until complete consumption of the intermediate material by TLC. Subsequently, the reaction was diluted with water and then extracted with EtOAc three times. Combined organic phases were washed with brine, dried over MgSO4, filtered and concentrated *in vacuo*. Purification by flash chromatography (20 g SiO2, 10:1 PE/EtOAc) gave a white amorphous solid (190 mg, 81% 2 steps).

^{1}H NMR (400 MHz, CDCl$_3$) δ 7.93-7.81 (m, 4H, Nap*H*), 7.58 (m, 1H, Nap*H*), 7.52–7.46 (m, 2H, Nap*H*), 7.02 (m, 2H, Ph*H*), 6.84 (m, 2H, Ph*H*), 5.10 (d, J = 10.8 Hz, 1H, C*H*$_2$), 5.01 (d, J = 10.9 Hz, 1H, C*H*$_2$), 4.71 (d, J = 8.5 Hz, 1H, *H-1*), 3.78 (s, 3H, -C*H*$_3$), 3.70 (dd, J = 9.7, 8.1 Hz, *H-2*), 3.36 (dd, J = 9.7, 9.2, 1H, *H-3*), 3.32 (m, 1H, *H-5*), 3.26 (m, 1H, *H-4*) 1.41 (d, J = 6.1 Hz, 3H, *H-6*) ppm.

^{13}C{^{1}H} NMR (101 MHz, CDCl$_3$) δ 155.7 (*Ar*-OMe), 151.0 (*Ar*-O-C-1) 134.7 (*Nap*-CH$_2$),133.3 (*Nap*), 128.3 (*Nap*), 128.1 (*Nap*), 127.7 (*Nap*), 127.4 (*Nap*), 126.2 (*Nap*), 126.2 (*Nap*), 126.1

(*Nap*), 118.8 (2C, Ar). 114.7 (2C, Ar), 101.4 (*C-1*), 81.1 (*C-3*), 75.5 (*C*H_2), 71.0 (*C-5*), 67.5 (*C-4*), 66.2 (*C-2*), 55.7 (*C*H_3O), 18.5 (*C-6*) ppm.

HRMS (ESI): m/z: $[M+NH_4]^+$ Calcd. for $C_{24}H_{28}N_7O_4$ 478.2197; found 478.2195.

Optical rotation: $[\alpha]_D^{20}$ = +66.8 (c 1.1, $CHCl_3$).

2.12 (4-Methoxy)phenyl 4-azido-4-deoxy-3-O-benzyl-6-O-triisopropylsilyl-β-D-mannopyranoside (S-10)

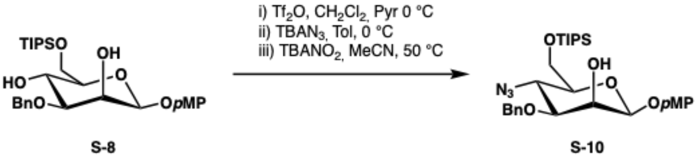

Compound **S-8** (445 mg, 0.835 mmol, 1.0 eq.) was dissolved in a 3:1 mix of CH_2Cl_2 (4.8 mL) and pyridine (1.6 mL). The solution was cooled to 0 °C using an ice bath, and Tf_2O (1 M in CH_2Cl_2, 2.51 mL, 2.51 mmol, 3.0 eq.) was added dropwise. The mixture was stirred for 3 h and poured onto 1 N HCl. The aqueous phase was extracted three times with CH_2Cl_2 and the combined organic phases were then washed with aq. sat. $NaHCO_3$. The organic phase was dried over $MgSO_4$, filtered and the solvent was evaporated, thus obtaining the ditriflated product. This was then dissolved in toluene (4.1 mL) and an ice bath was used to cool the reaction to 0 °C. $TBAN_3$ (250 mg, 0.835 mmol, 1.0 eq.) was added, and the reaction was warmed to 25 °C over 16 h. Subsequently the solvent was evaporated and the remaining oil was dissolved in acetonitrile (1.8 mL) and $TBANO_2$ (298 mg, 1.00 mmol, 1.2 eq.) was added. The reaction mixture was heated to 50 °C for 5 h and subsequently the solvent was evaporated *via* reduced pressure. The crude product was purified via column chromatography (40 g SiO_2, 5:1 PE/EtOAc) to yield **S-10** as a pale orange solid (120 mg, 26 %).

^{1}H NMR (600 MHz, $CDCl_3$) δ 7.46 – 7.31 (m, 5H, *H*Ar), 7.00 (m, 2H, *H*Ar), 6.78 (m, 2H, *H*Ar), 4.82 (d, *J* = 1.0 Hz, 1H, *H-1*), 4.81 (d, *J* = 11.5 Hz, 1H, C*H_2*), 4.73 (d, *J* = 11.9 Hz, 1H, C*H_2*), 4.27 (dd, *J* = 3.5, 1.2 Hz, 1H, *H-2*), 4.00 (dd, *J* = 11.2, 1.9 Hz, 1H, *H-6a*), 3.92 (dd, *J* = 6.2, 5.4 Hz, 1H, *H-6b*) 3.90 (dd, *J* = 10.0, 10.0 Hz, 1H, *H-4*), 3.76 (s, 3H, -OC*H_3)*, 3.51 (dd, *J* = 9.8, 3.0 Hz, 1H, *H-3*), 3.20 (ddd, *J* = 10.3, 5.4, 1.9 Hz, 1H, *H-5*), 2.49 (d, *J* = 2.6 Hz, 1H, O**H**), 1.16 – 1.01 (m, 21H, *TIPS*) ppm.

^{13}C{^{1}H} NMR (151 MHz, $CDCl_3$) δ 155.3 (*Ar*-OMe), 151.0 (*Ar*-O-C-1) 137.1 (*Ar*-CH_2),128.7 (2C, Ar), 128.2 (*Ar*), 128.2 (2C*, Ar*), 118.4 (2C *, Ar*), 114.4 (2C*, Ar*), 99.2 (C-1), 80.0 (C-3), 76.0 (C-5), 71.5 (CH_2), 67.6 (C-2), 63.2 (C-6), 57.7 (C-4), 55.6 (CH_3O), 18.0 (TIPS), 17.9 (TIPS), 11.9 (TIPS) ppm.

HRMS (ESI) m/z: $[M+Na]^+$ Calcd. for $C_{29}H_{43}N_3NaO_6Si$ 580.2813; found 580.2837.

2.13 (4-Methoxy)phenyl 2,4-diazido-2,4-deoxy-3-O-benzyl-6-O-triisopropylsilyl-β-D-glucopyranoside (6e)

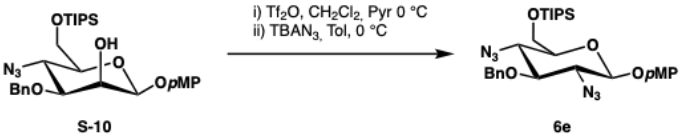

Monoazide **S-10** (92 mg, 0.165 mmol, 1.0 eq.) was dissolved in CH_2Cl_2 (0.9 mL) and pyridine (0.3 mL). The solution was cooled to 0 °C using an ice bath, and Tf_2O (1 M in CH_2Cl_2, 0.2 mL, 0.198 mmol, 1.2 eq) was added dropwise. The mixture was stirred for 2 h and poured onto 1N HCl. The aqueous phase was extracted three times with CH_2Cl_2, and the combined organic phases was washed with aq. sat. $NaHCO_3$. The organic phase was then dried over $MgSO_4$, filtrated and the solvent was evaporated. The residue was dissolved in toluene (0.8 mL) and $TBAN_3$ (59 mg, 0.198 mmol, 1.2 eq.) was added. The solution was stirred for 18 h and subsequently the solvent was evaporated. The crude product was purified using column chromatography (17 g, 15:1 PE/EE) to yield the product as a colorless amorphous solid (51 mg, 53 %).

^{1}H NMR (400 MHz, $CDCl_3$) δ 7.49 – 7.28 (m, 5H, *H*Ar), 7.04 (m, 2H, *H*Ar), 6.81 (m, 2H, *H*Ar), 4.93 (d, *J* = 10.4 Hz, 1H, C*H₂*), 4.85 (d, *J* = 10.5 Hz, 1H, C*H₂*), 4.66 (d, *J* = 8.1 Hz, 1H, *H-1*), 4.00 (dd, *J* = 11.3, 1.9 Hz, 1H, *H-6a*), 3.93 (dd, *J* = 11.3, 4.3 Hz, 1H, *H-6b*), 3.78 (s, 3H, C*H₃*O), 3.68 (dd, *J* = 9.8, 9.8 Hz, 1H, *H-4*), 3.64 (dd, *J* = 9.5, 8.1 Hz, 1H, *H-2*), 3.33 (dd, *J* = 9.8, 9.8 Hz, 1H, *H-3*), 3.22 (ddd, *J* = 10.1, 4.4, 1.9 Hz, 1H, *H-5*), 1.16 – 1.01 (m, 21H, *TIPS*) ppm.

^{13}C{^{1}H} NMR (101 MHz, $CDCl_3$) δ 155.8 (*Ar*-OMe), 151.1 (*Ar*-O-C-1) 137.2 (*Ar*-CH_2),128.6 (2C, *Ar*), 128.6 (2C, *Ar*), 128.2 (*Ar*), 119.1 (2C, *Ar*), 114.5 (2C, *Ar*), 102.0 (*C-1*), 81.4 (*C-3*), 75.7 (*C-5*), 75.6 (CH_2), 66.0 (*C-2*), 62.8 (*C-6*), 61.4 (*C-4*), 55.7 (CH_3O), 17.9 (*TIPS*), 17.9 (*TIPS*), 11.9 (*TIPS*) ppm.

HRMS (ESI) m/z: $[M+Na]^+$ Calcd. for $C_{29}H_{42}N_6NaO_5Si$ 605.2878; found 605.2878.

Optical rotation: $[\alpha]_D^{20}$ = +29,2 (1.9 $CHCl_3$).

3 Equatorially selective mono azide reduction

3.1 General procedure

Diazido pyranoside (0.07 mmol, 1.0 eq.) was added to a round bottom flask and dissolved in a 1:1 mix of methanol and pyridine (0.05 M combined). Subsequently, Lindlar catalyst was added (0.0035 mmol, 0.05 eq.) and the flask was evacuated and backfilled with hydrogen from

a balloon three times. The reaction was then stirred under an atmosphere of hydrogen until TLC analysis revealed full conversion (2-16 h). Subsequently, the reaction was filtered, the solvent removed and the crude product was purified by flash chromatography to reveal the desired product.

3.2 (4-Methoxy)phenyl 2,4-dideoxy-2-azido-3-O-benzyl-4-amino-6-O-triisopropylsilyl-β-D-mannopyranoside (7a)

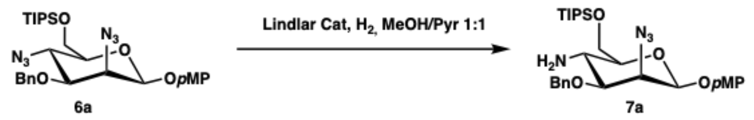

Product synthesized according to the general procedure. Reduction under H_2 atmosphere was accomplished after 12 h. Product obtained was an offwhite amorphous solid.

Purification 3 g SiO_2, 3:2 PE/EtOAc

Yield 32 mg (82%).

^{1}H NMR (400 MHz, $CDCl_3$) δ 7.45 – 7.31 (m, 5H, *Ph*CH_2-), 6.99 (m, 2H, *Ph*OMe), 6.80 (m, 2H, *Ph*OMe), 4.97 (d, J = 1.2 Hz, 1H, *H-1*), 4.79 (d, J = 11.1, 1H, -*CH*$_2$-), 4.61 (d, J = 11.4, 1H, -C*H*$_2$-), 4.12 (dd, J= 3.5, 1.1 Hz, 1H, *H-2*), 3.95 (dd, J = 4.8, 1.1, 2H, *H-6*), 3.77 (s, 3H, -O*Me*), 3.53 (dd, J = 9.9, 3.4 Hz, 1H, *H-3*), 3.37 (dddd J = 9.8, 4.8, 1H, *H-5*), 3.22 (dd, J = 9.9, 9.6 Hz, 1H, *H-4*) 1.05 (m, 21H, *TIPS*) ppm.

^{13}C{^{1}H} NMR (101 MHz, $CDCl_3$) δ 155.3 (*Ar*-OMe), 150.8 (*Ar*-O-C-1), 137.1 (*Ar*-CH_2), 128.8 (*Ar*), 128.4 (*Ar*), 128.3 (*Ar*), 118.1 (*Ar*), 114.5 (*Ar*), 98.7 (*C-1*), 81.3 (*C-3*), 71.9 (*C-5*), 65.0 (-C*H*$_2$-), 60.1 (*C-2*), 55.6 (-O*Me*), 50.3 (*C-4*), 18.0 (-*TIPS*), 11.8 (-*TIPS*) ppm.

HRMS (ESI) m/z: $[M+H]^+$ Calcd. for $C_{29}H_{45}N_4O_5Si$ 557.3154; found 557.3153.

3.3 (4-Methoxy)phenyl 2-amino-4-azido-2,4-deoxy-3-O-benzyl-β-D-fucopyranoside (7b)

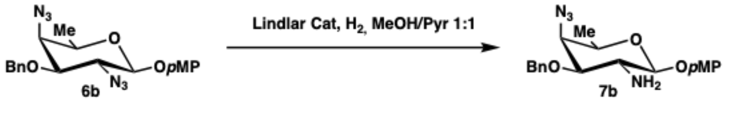

Product synthesized according to the general procedure. Reduction under H_2 atmosphere was accomplished after 4h. Product obtained was an offwhite amorphous solid.

Purification 3 g SiO_2, 100% EtOAc

Yield 20 mg (74%)

^{1}H NMR (400 MHz, $CDCl_3$) δ 7.43-7.30 (m, 5H, *Ph*), 6.99 (m, 2H, *Ph*OMe), 6.80 (m, 2H, *Ph*OMe), 4.80 (d, J = 11.2, 1H, -C*H*$_2$-), 4.64 (d, J= 8.3, 1H, *H-1*), 4.59 (d, J = 11.2, 1H, -C*H*$_2$-), 3.79-3.71 (m, 1H, *H-4*), 3.75 (s, 3H, O*Me*), 3.70-3.64 (m, 1H, *H-5*), 3.65-3.51 (m, 1H, *H-3*), 2.48 (b, 2H, -N*H*$_2$), 1.36 (d, J = 6.1 Hz, 3H, *H-6*) ppm.

^{13}C{^{1}H} NMR (101 MHz, $CDCl_3$) δ 155.4 (*Ph*-OMe), 151.2 (*Ph*-O-C-1), 137.0 (*Ph*CH$_2$-), 128.7 (*Ph*CH$_2$-), 128.4 (*Ph*CH$_2$-), 128.3 (*Ph*CH$_2$-), 118.8 (*Ar*), 114.4 (*Ar*), 103.3 (*C-1*), 81.9 *(C-3*), 72.2 (-*CH*$_2$-), 69.5 *(C-5*), 61.1 *(C-4*), 55.6 *(-OMe*), 52.4 *(C-2*) 17.8 *(C-6*) ppm.

HRMS (ESI) m/z: $[M+Na]^+$ Calcd. for $C_{20}H_{24}N_4NaO_4$ 407.1690; found 407.1696.

3.4 (4-Methoxy)phenyl 2-amino-4-azido-2,4-dideoxy-3-O-benzyl-β-D-galactopyranoside (7c)

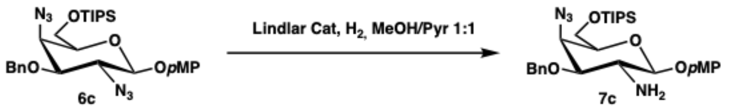

Product synthesized according to the general procedure. Reduction under H_2 atmosphere was accomplished after 5 h. Product obtained was a white amorphous solid.

Purification 4 g SiO_2, 3:2 PE/EtOAc

Yield 31 mg (75%)

^{1}H NMR (400 MHz, $CDCl_3$) δ 7.44-7.31 (m, 5H, *Ph*CH$_2$-) 6.98 (m, 2H, *Ph*OMe), 6.79 (m, 2H, *Ph*OMe) 4.81 (d, J = 11.3 Hz, 1H, -*CH*$_2$-) 4.64 (d, J = 7.84 Hz, 1H, *H-1*) 4.64 (d, J = 11.4 Hz, 1H, -*CH*$_2$-), 4.06 (dd, J = 3.2, 1.1 Hz, 1H, *H-4*) 3.86 (m, 2H, *H-6*), 3.76 (s, 3H, -O*Me*), 3.60 (dd, J = 10.0, 3.4 Hz, 1H, *H-3*), 3.56 (ddd, J= 7.7, 5.8, 1.4 Hz, 1H, *H-5*) 3.43 (dd, J = 10.0, 7.9 Hz, 1H, *H-2*) 1.92 (b, 2H, -N*H*$_2$), 1.16-1.02 (m, 21H) ppm.

^{13}C{^{1}H} NMR (101 MHz, $CDCl_3$) δ 155.4 (*Ar*-OMe), 151.2 (*Ar*-O-C-1), 137.1 (*Ar*-CH$_2$), 128.8 (*Ar*), 128.4 (*Ar*), 128.3 (*Ar*) , 118.8 (*Ar*), 114.4 (*Ar*), 103.7 (*C-1*), 81.8 (*C-3*), 73.7 (*C-5*), 72.3 (-*C*H$_2$-), 62.0 (*C-5*), 57.3 (*C-4*), 55.6 (-O*Me*), 52.9 (*C-2*), 18.0 (*TIPS*), 17.9 (*TIPS*), 11.8 (*TIPS*) ppm.

HRMS (ESI) m/z: $[M+Na]^+$ Calcd. for $C_{29}H_{44}N_4NaO_5Si$ 579.2973; found 579.2979.

4 HSQC spectra of compounds 6 and 7

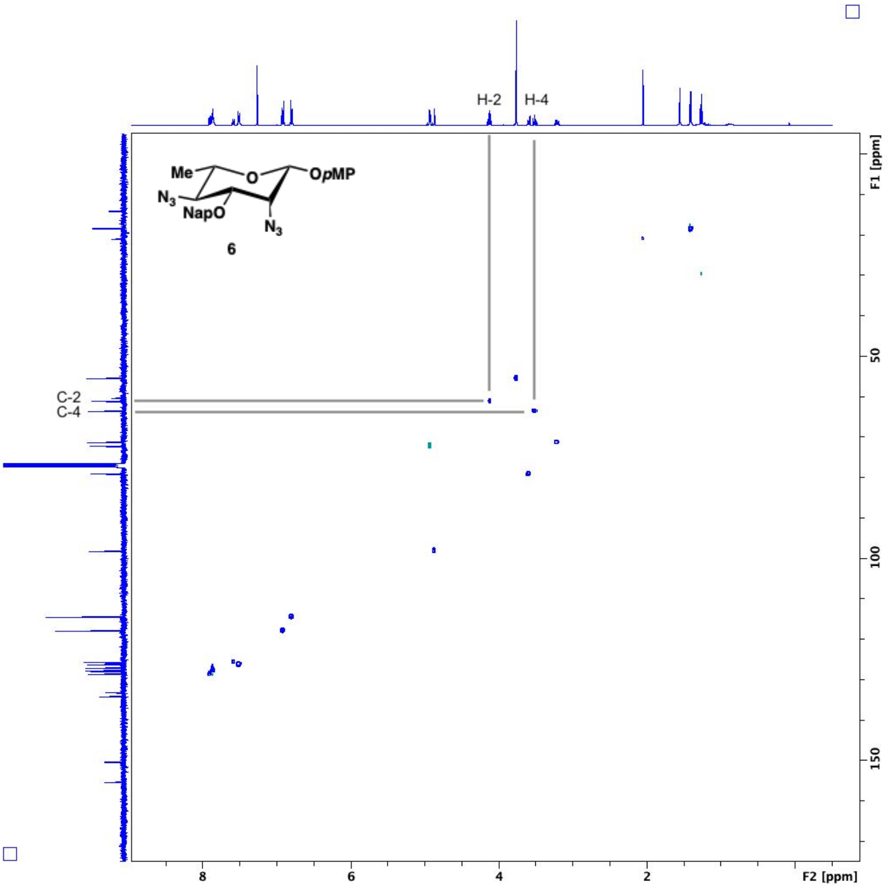

400 MHz

$CDCl_3$

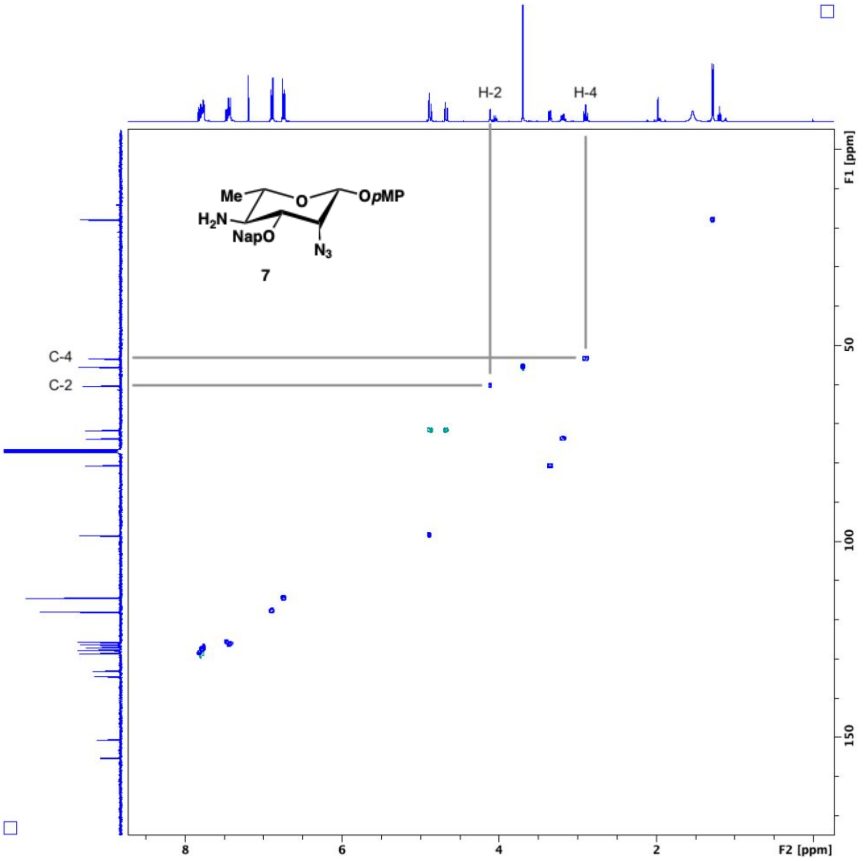

400 MHz

$CDCl_3$

5 References

[1] Mandal, P. K. Convergent Synthesis of the Pentasaccharide Repeating Unit of the O-Antigen of Escherichia Coli O36. *Synthesis* **2015**, *47* (6), 836–844. https://doi.org/10.1055/s-0034-1379952.

[2] Di Gaetano, S.; Pirone, L.; Galdadas, I.; Traboni, S.; Iadonisi, A.; Pedone, E.; Saviano, M.; Gervasio, F. L.; Capasso, D. Design, Synthesis, and Anticancer Activity of a Selenium-Containing Galectin-3 and Galectin-9N Inhibitor. *International Journal of Molecular Sciences* **2022**, *23* (5), 2581. https://doi.org/10.3390/ijms23052581.

[3] Pero, B.; Peczuh, M. W. Synthesis of a Diacetonide-Protected, Mannose-Based Oxepine: Configurational Control of Anomeric Acetate Activation. *J. Org. Chem.* **2022**, *87* (11), 7474–7479. https://doi.org/10.1021/acs.joc.2c00206.

6 ^{1}H and ^{13}C Spectra

5 ^{1}H NMR (400 MHz, $CDCl_3$)

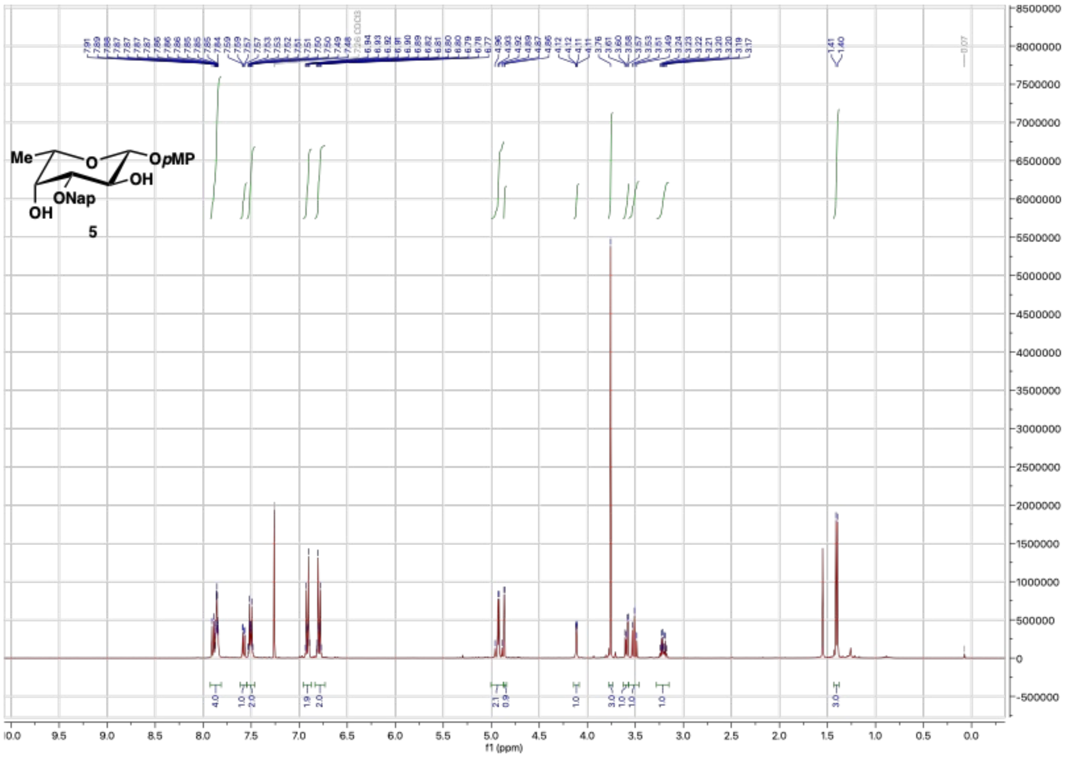

^{13}C{^{1}H} NMR (101 MHz, $CDCl_3$)

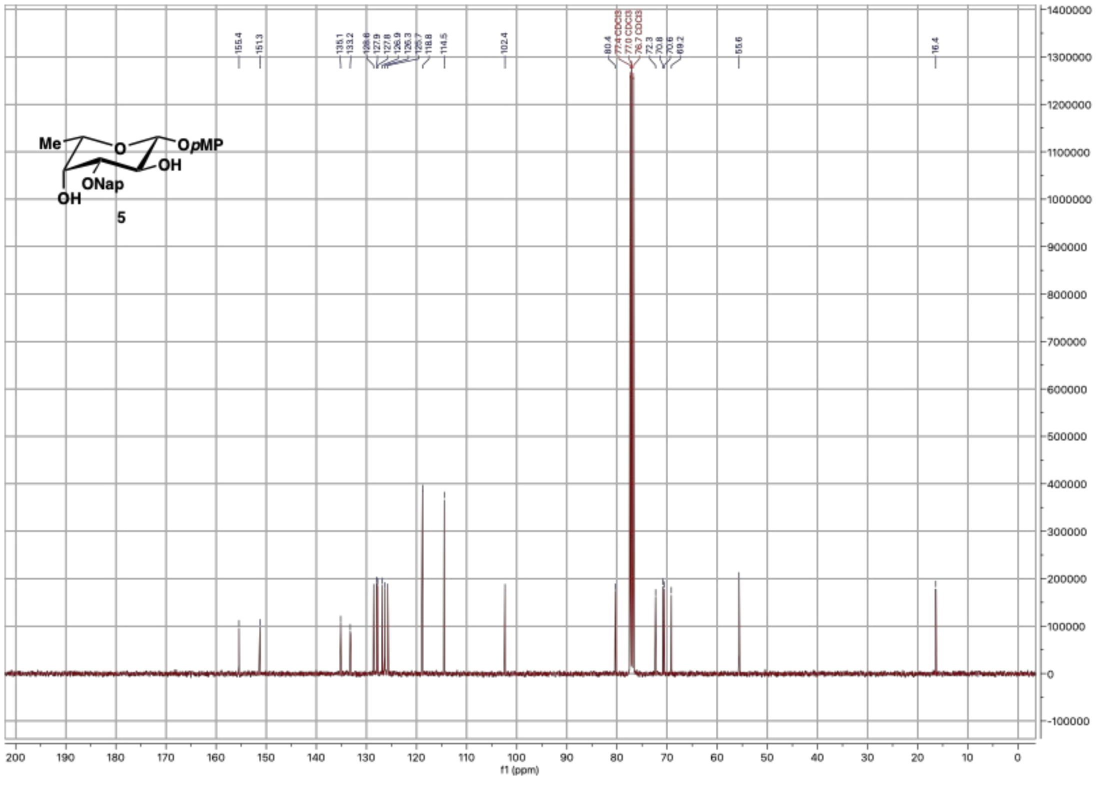

6

^{1}H NMR (400 MHz, $CDCl_3$)

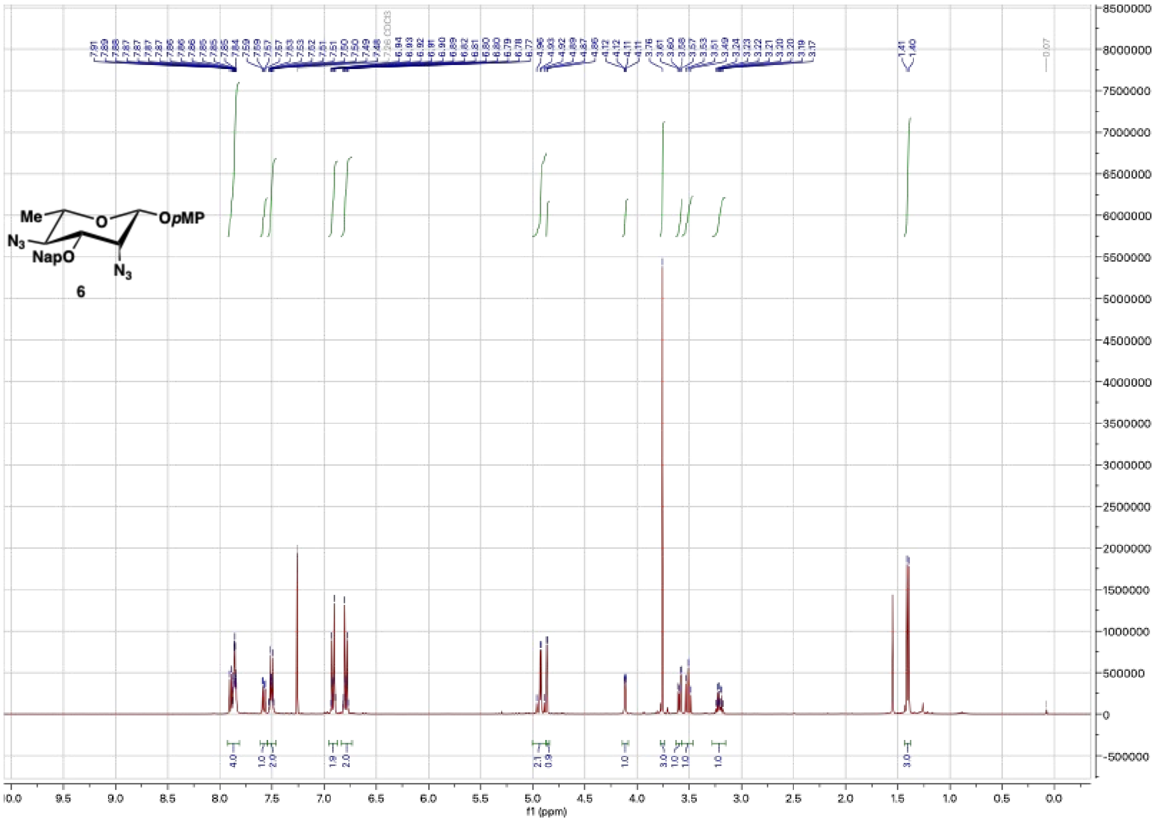

^{13}C{^{1}H} NMR (101 MHz, $CDCl_3$)

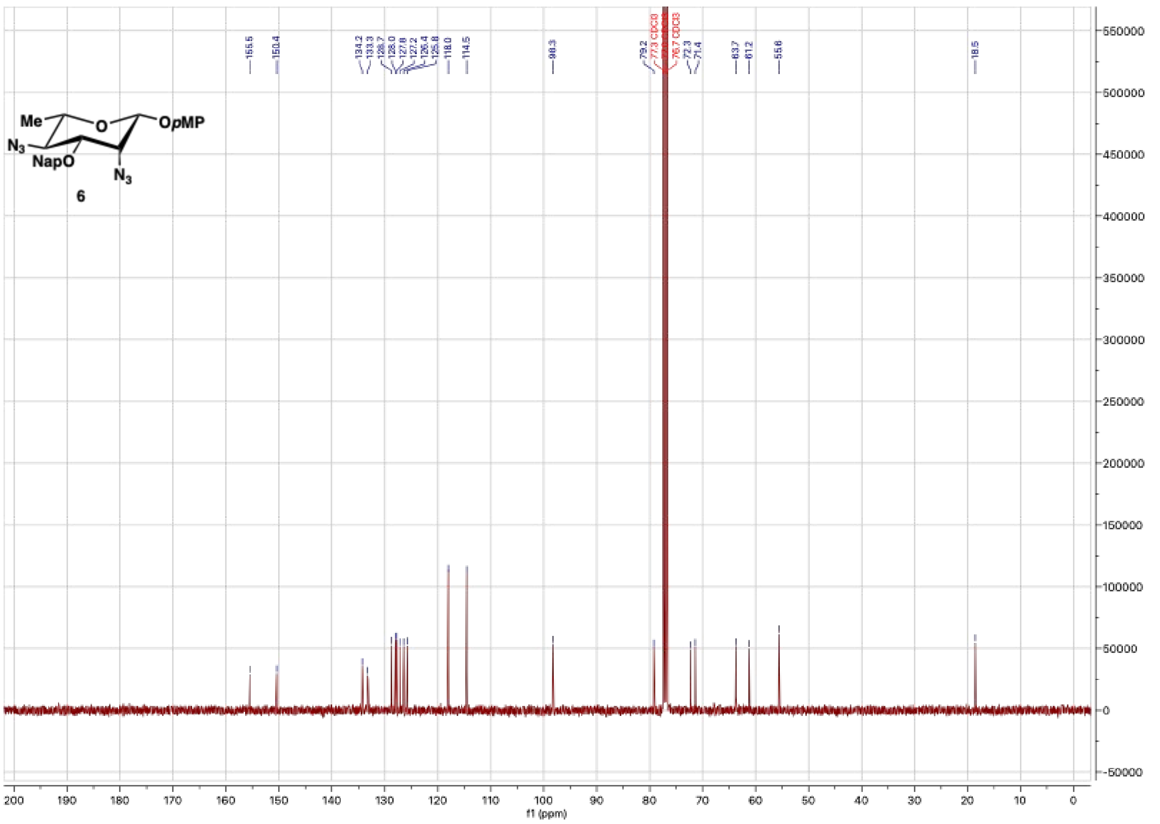

S-2

^{1}H NMR (400 MHz, $CDCl_3$)

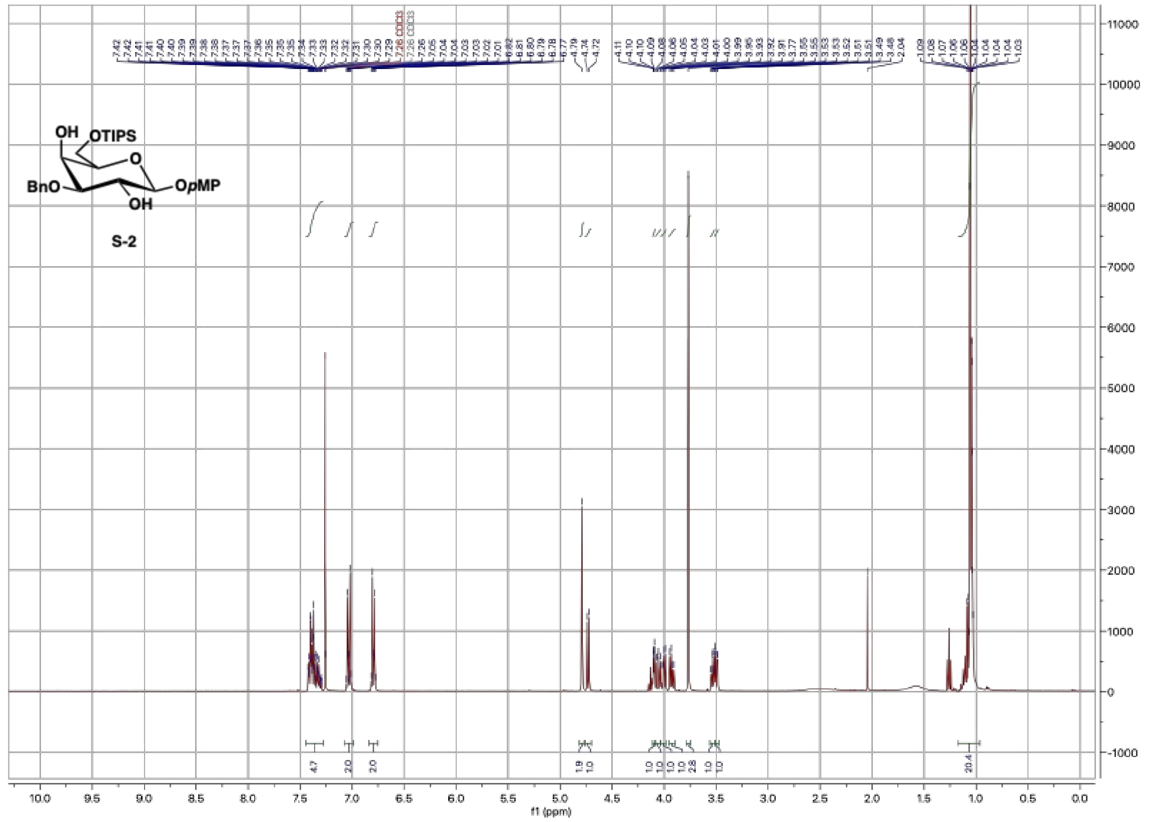

^{13}C{^{1}H} NMR (101 MHz, $CDCl_3$)

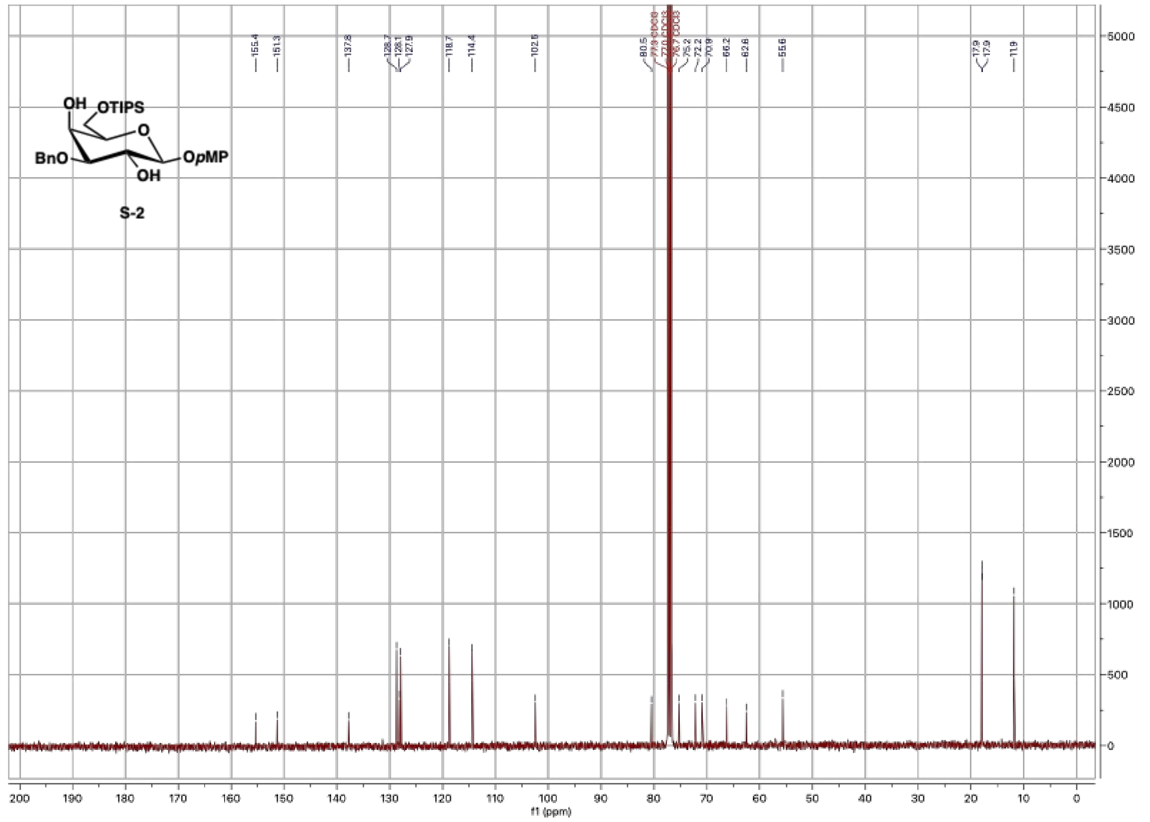

6a

^{1}H NMR (400 MHz, $CDCl_3$)

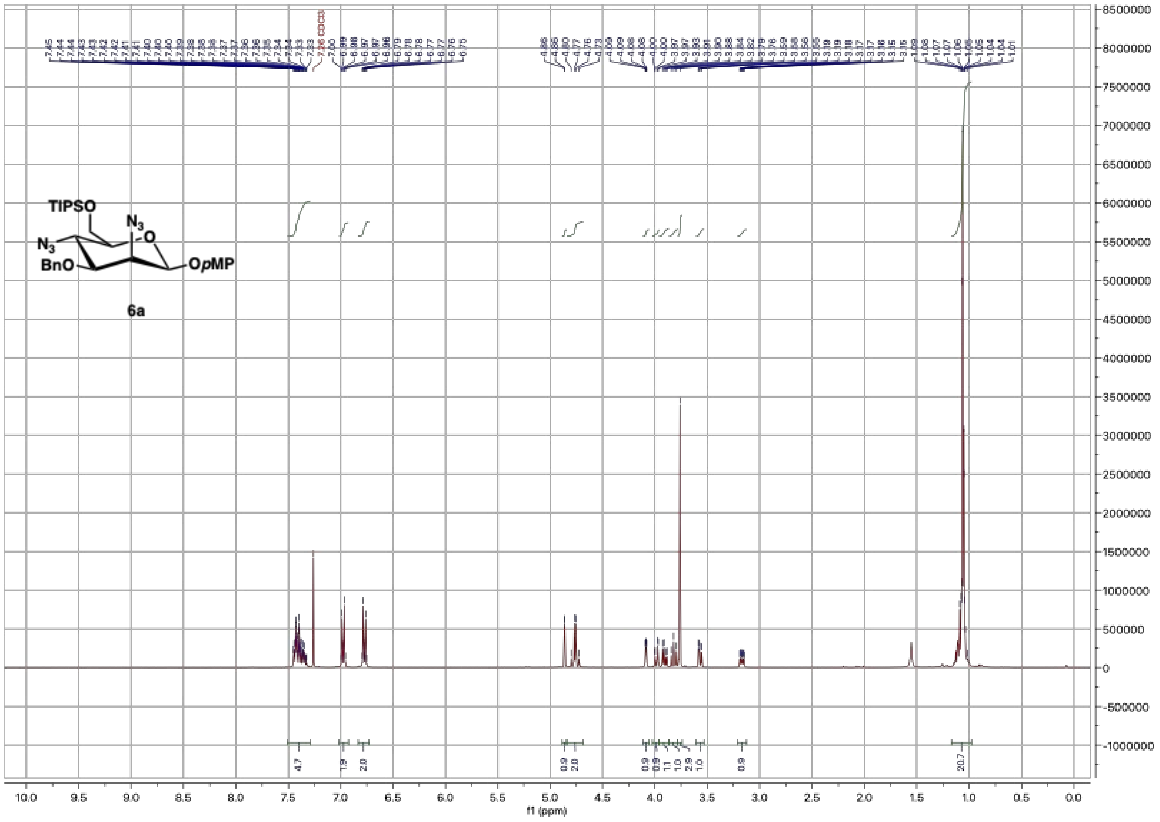

^{13}C{^{1}H} NMR (101 MHz, $CDCl_3$)

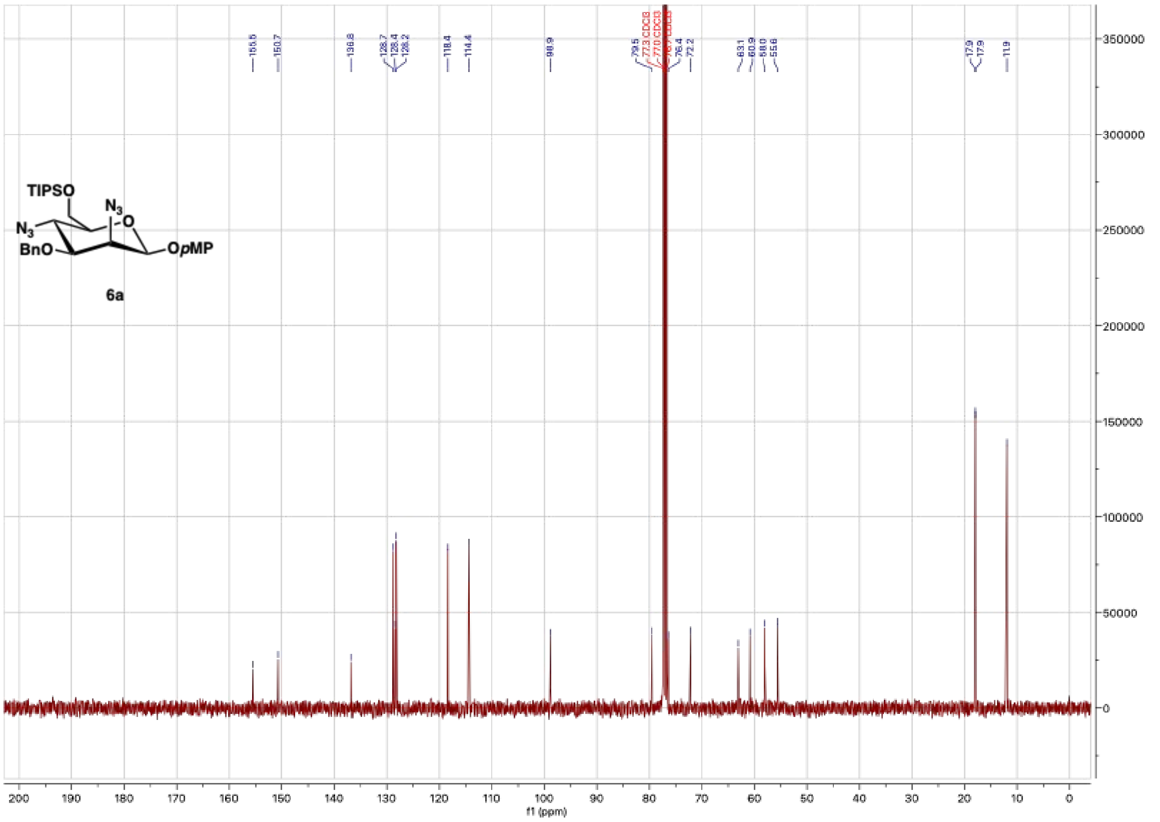

S-4

^{1}H NMR (400 MHz, d4-methanol)

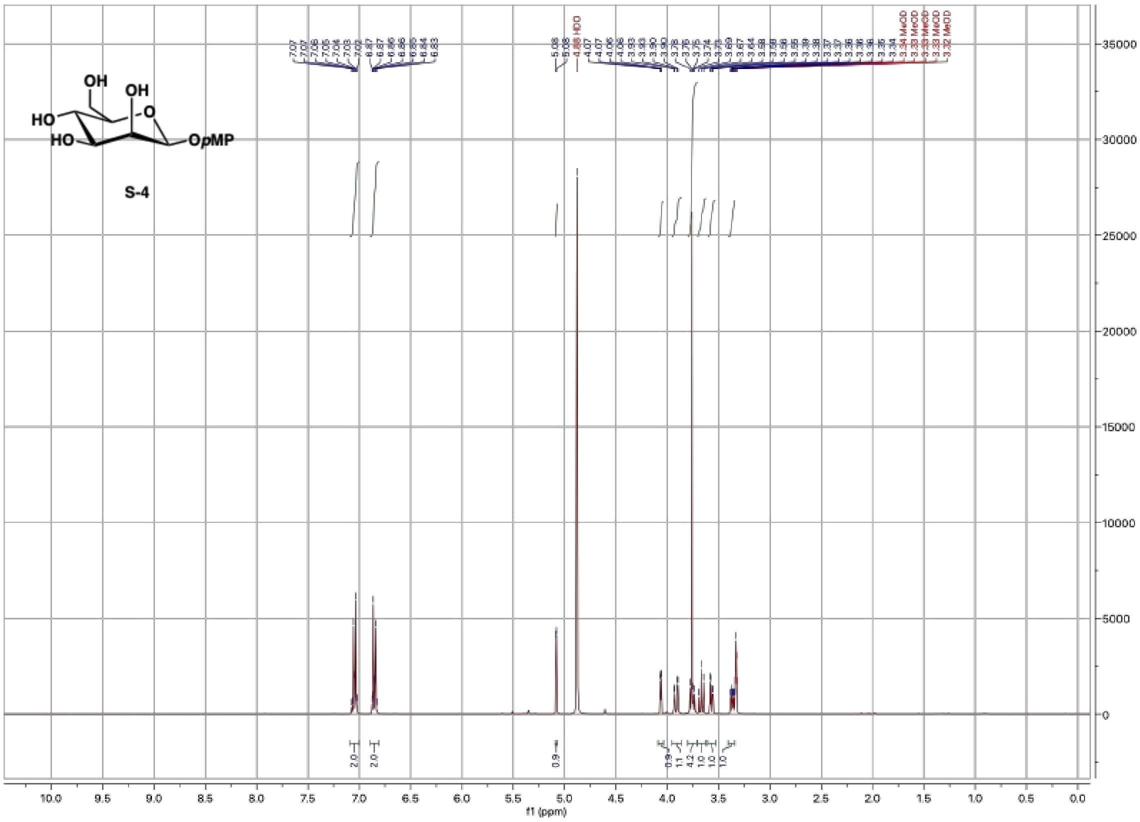

^{13}C{^{1}H} NMR (101 MHz, d4-methanol)

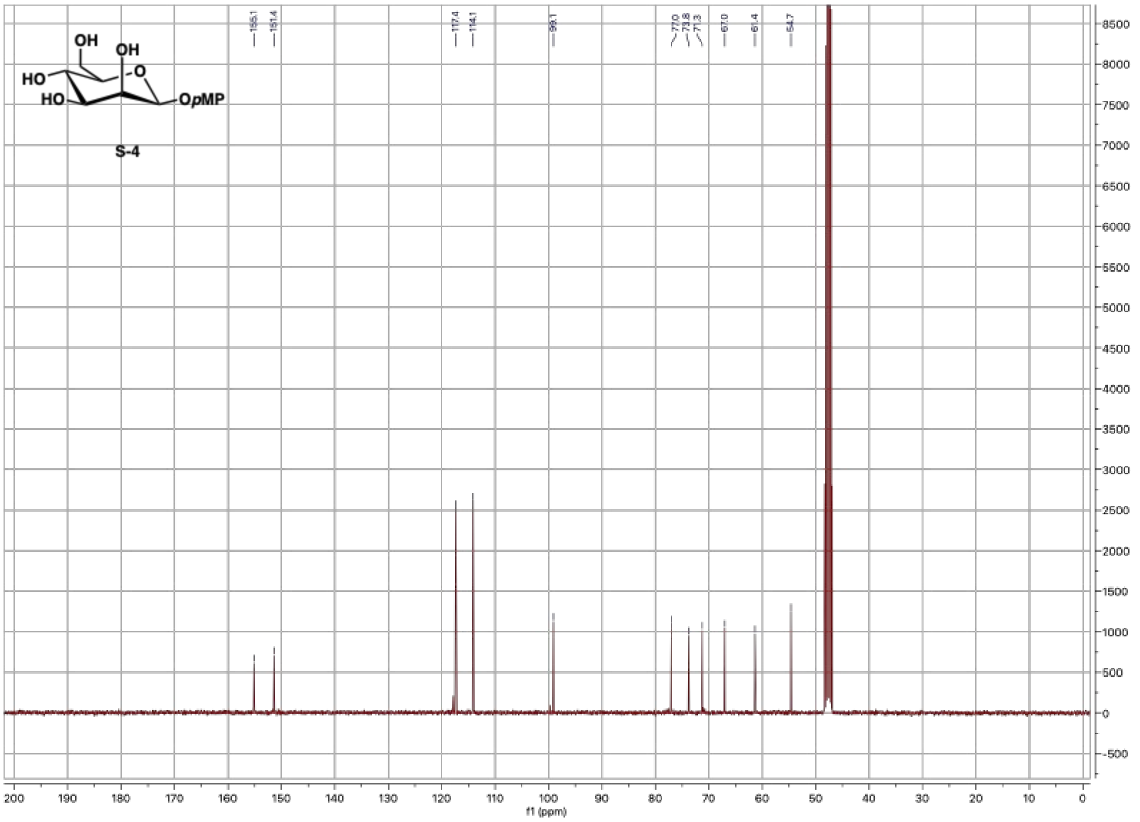

S-5

^{1}H NMR (400 MHz, d4-methanol)

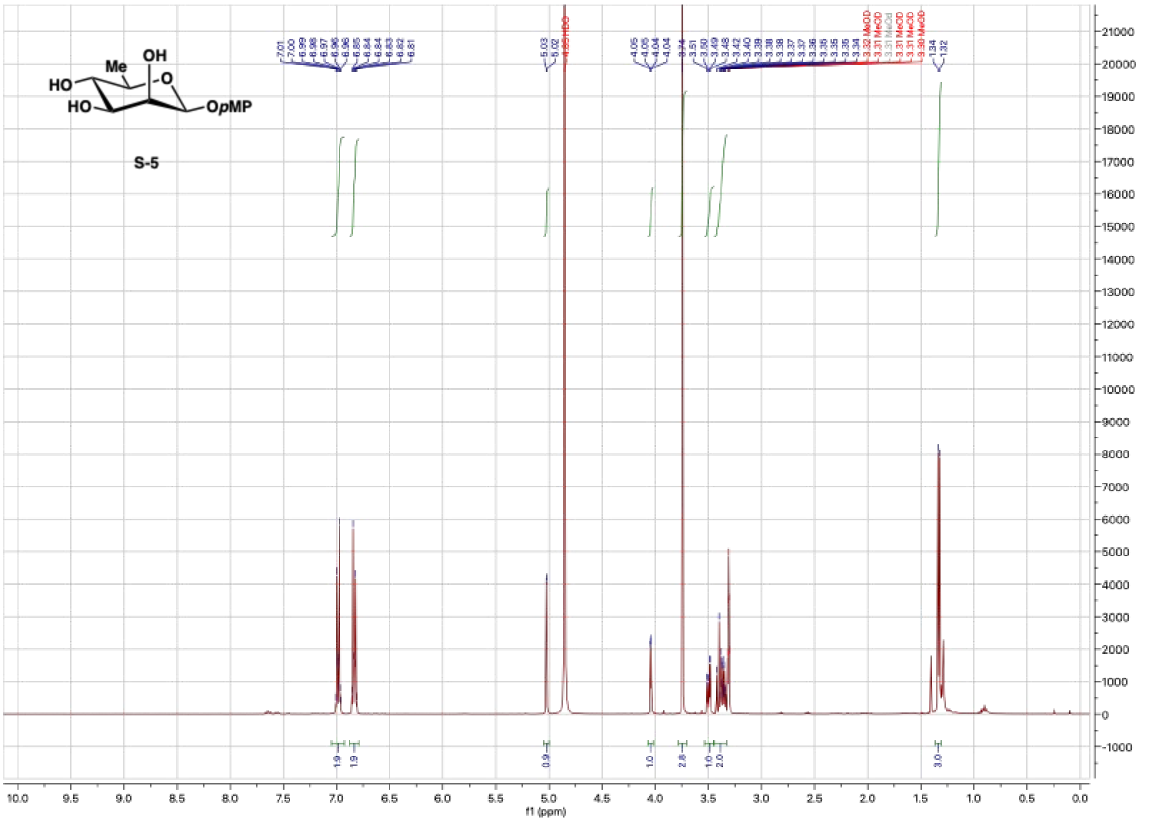

^{13}C{^{1}H} NMR (101 MHz, d4-methanol)

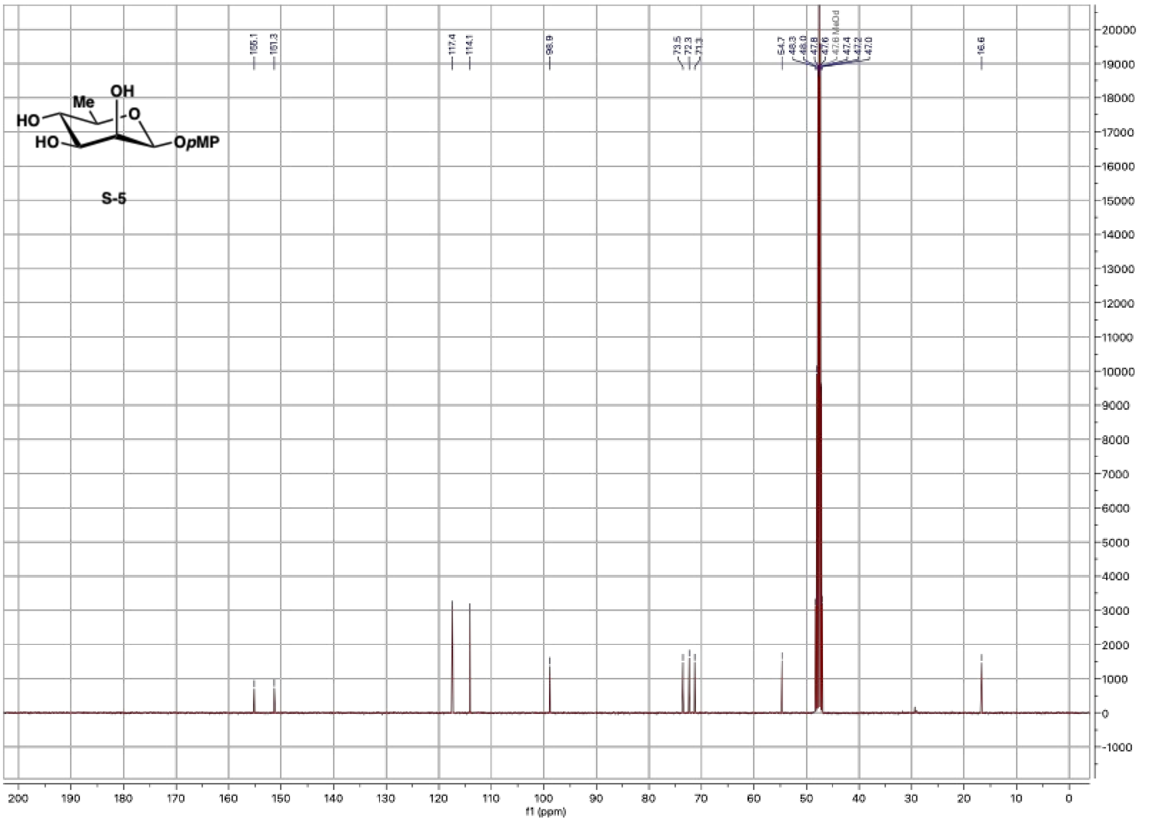

S-6

^{1}H NMR (400 MHz, $CDCl_3$)

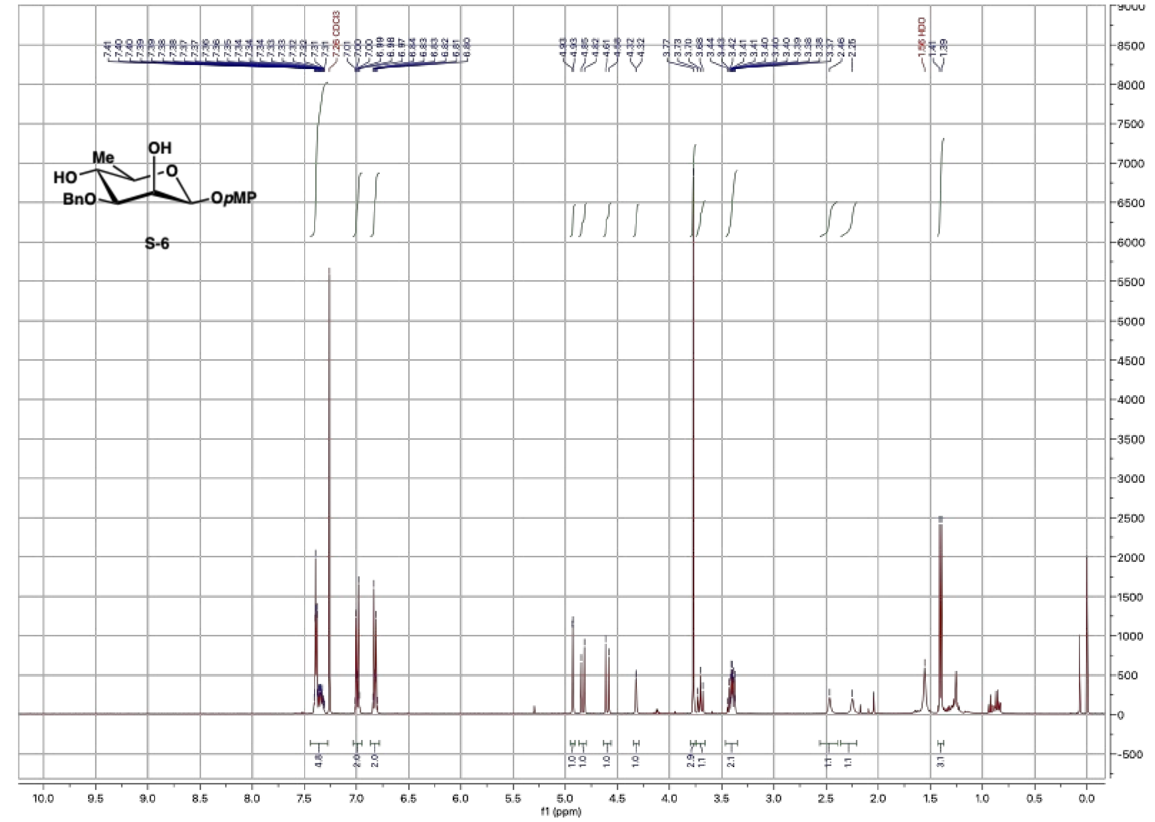

^{13}C{^{1}H} NMR (101 MHz, $CDCl_3$)

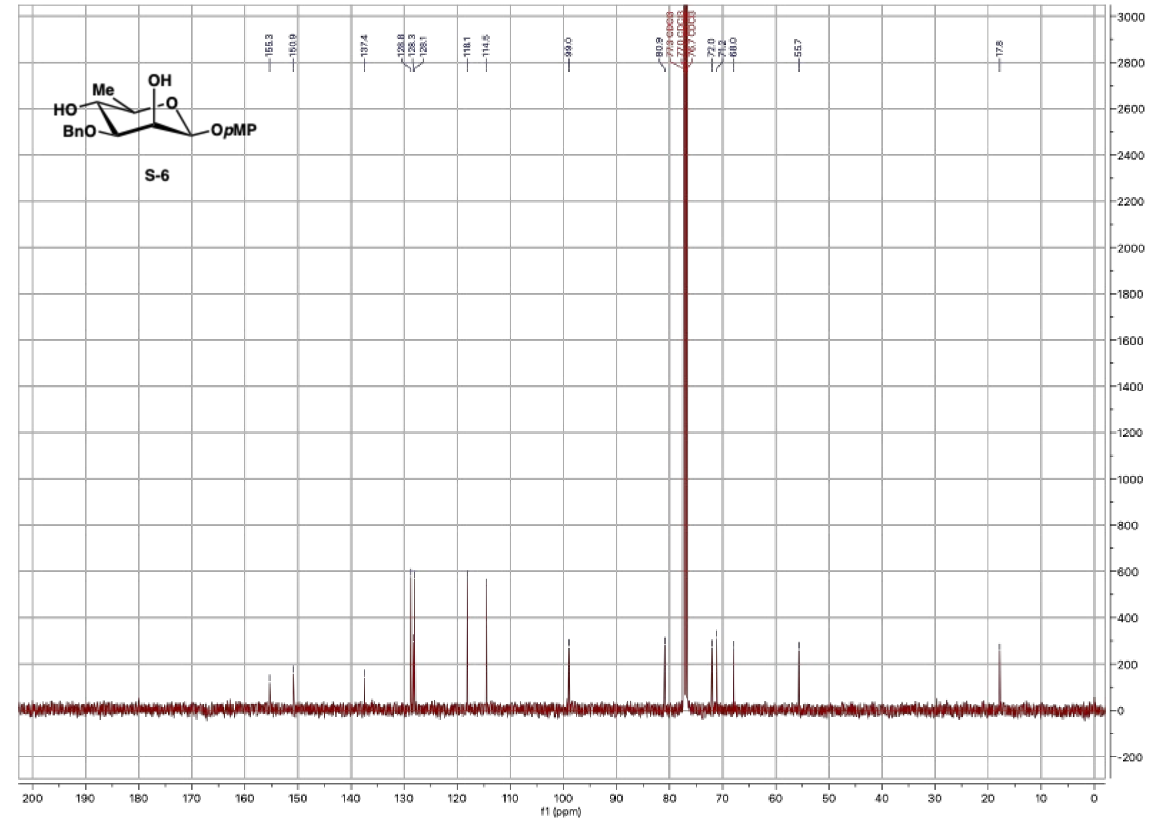

6b

^{1}H NMR (600 MHz, $CDCl_3$)

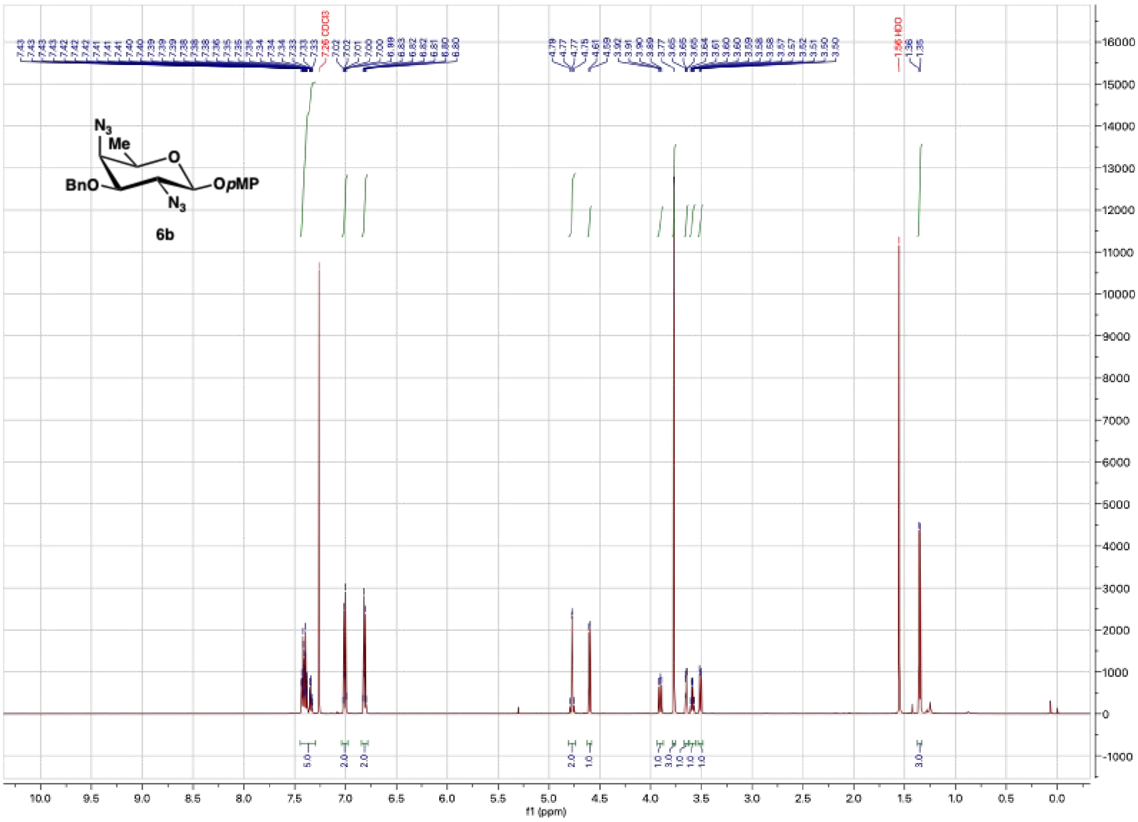

^{13}C{^{1}H} NMR (151 MHz, $CDCl_3$)

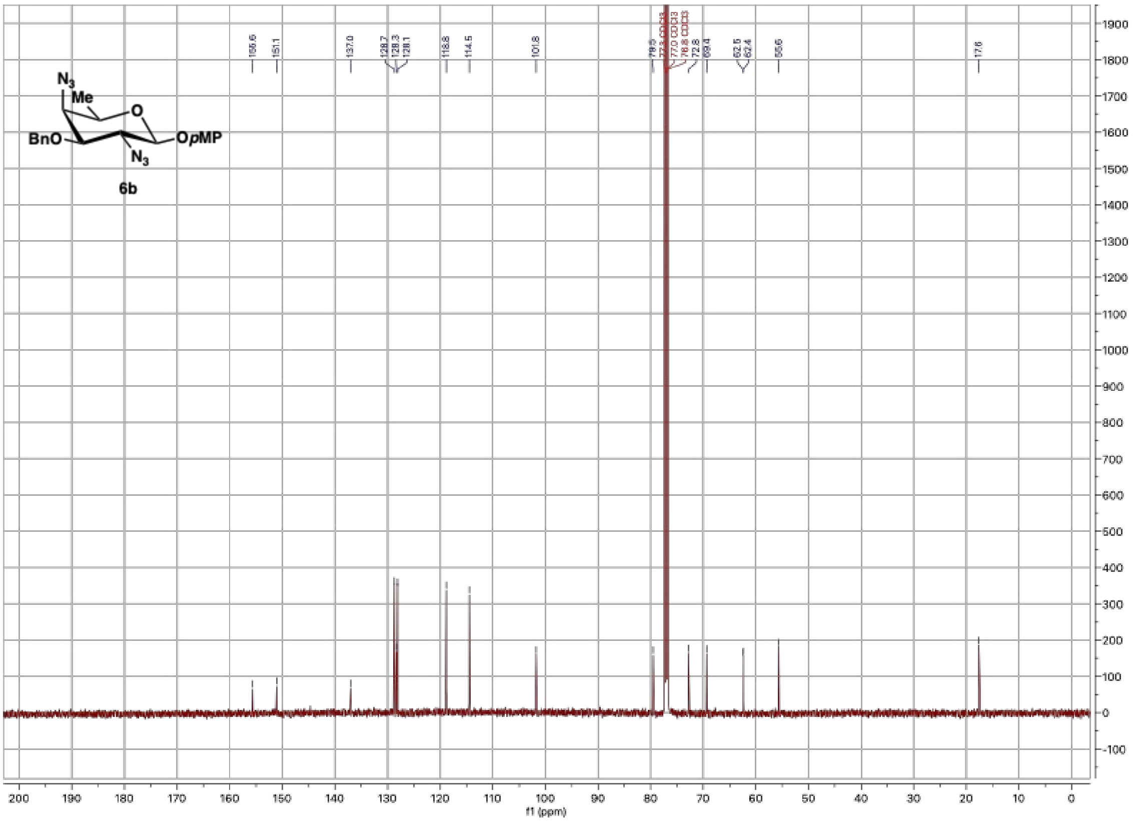

S-8

^{1}H NMR (600 MHz, $CDCl_3$)

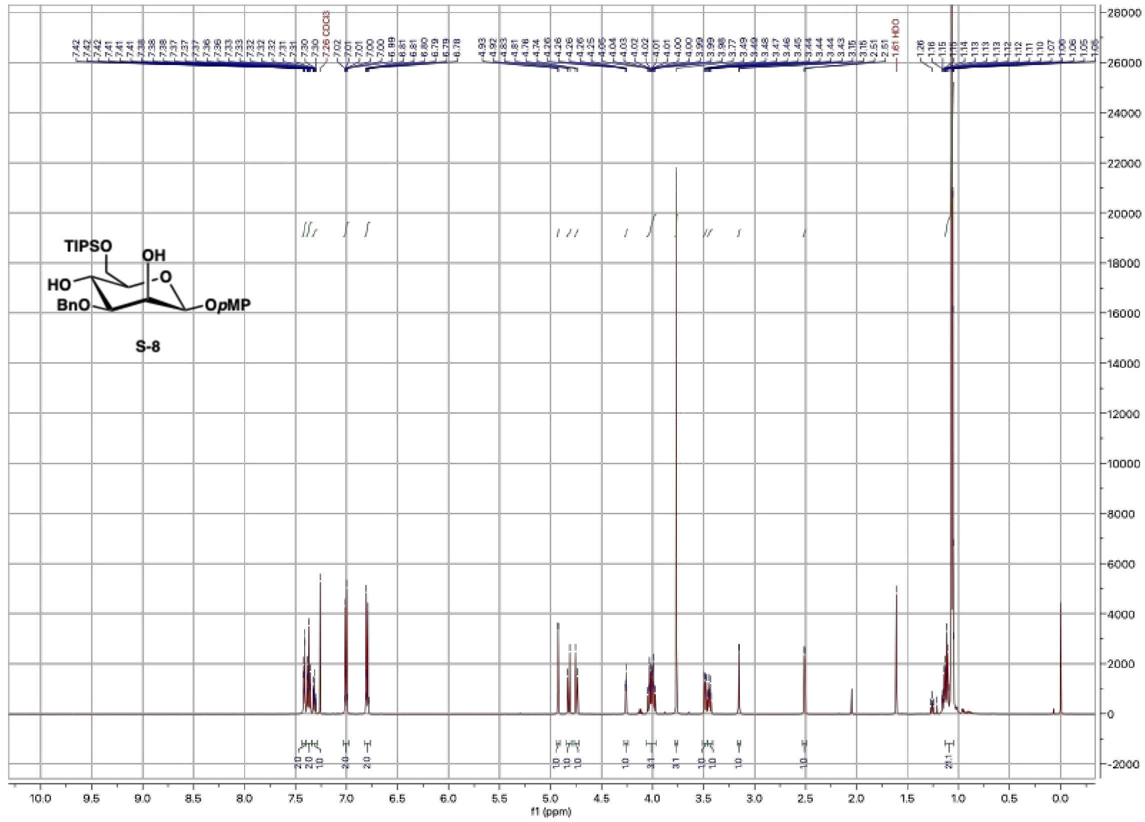

^{13}C{^{1}H} NMR (151 MHz, $CDCl_3$)

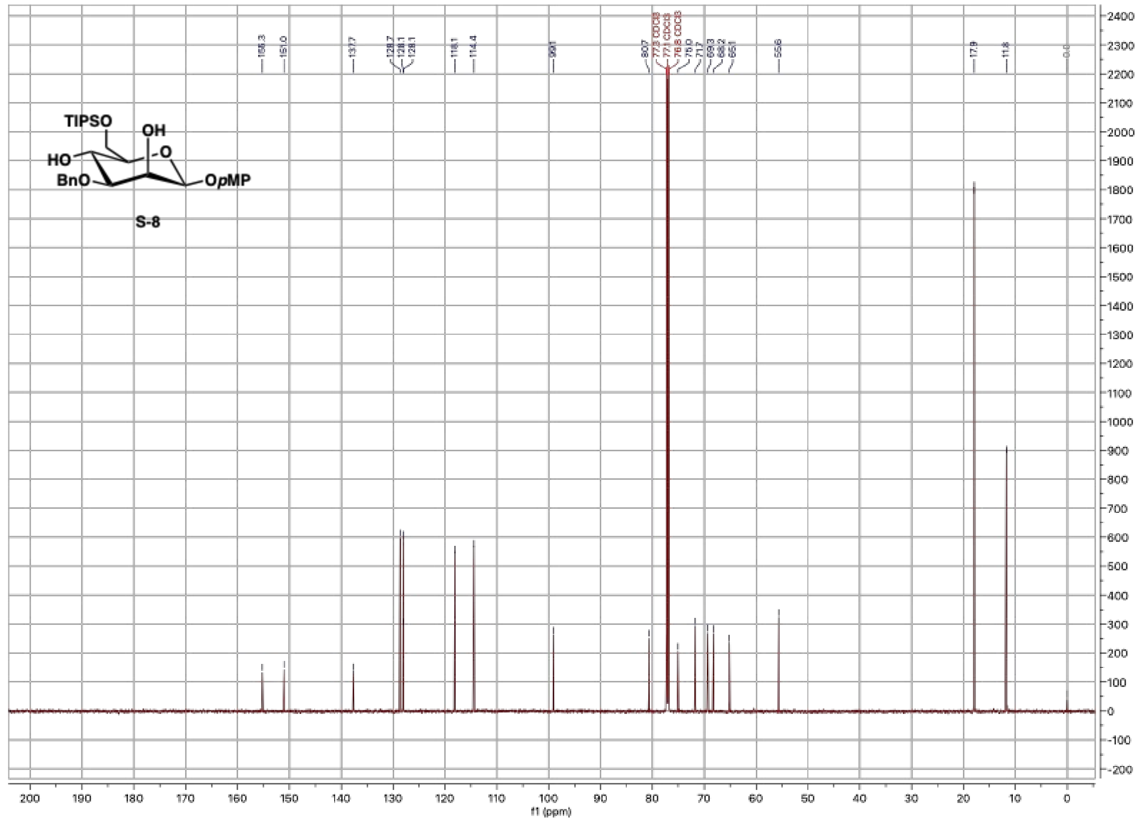

6c

^{1}H NMR (600 MHz, $CDCl_3$)

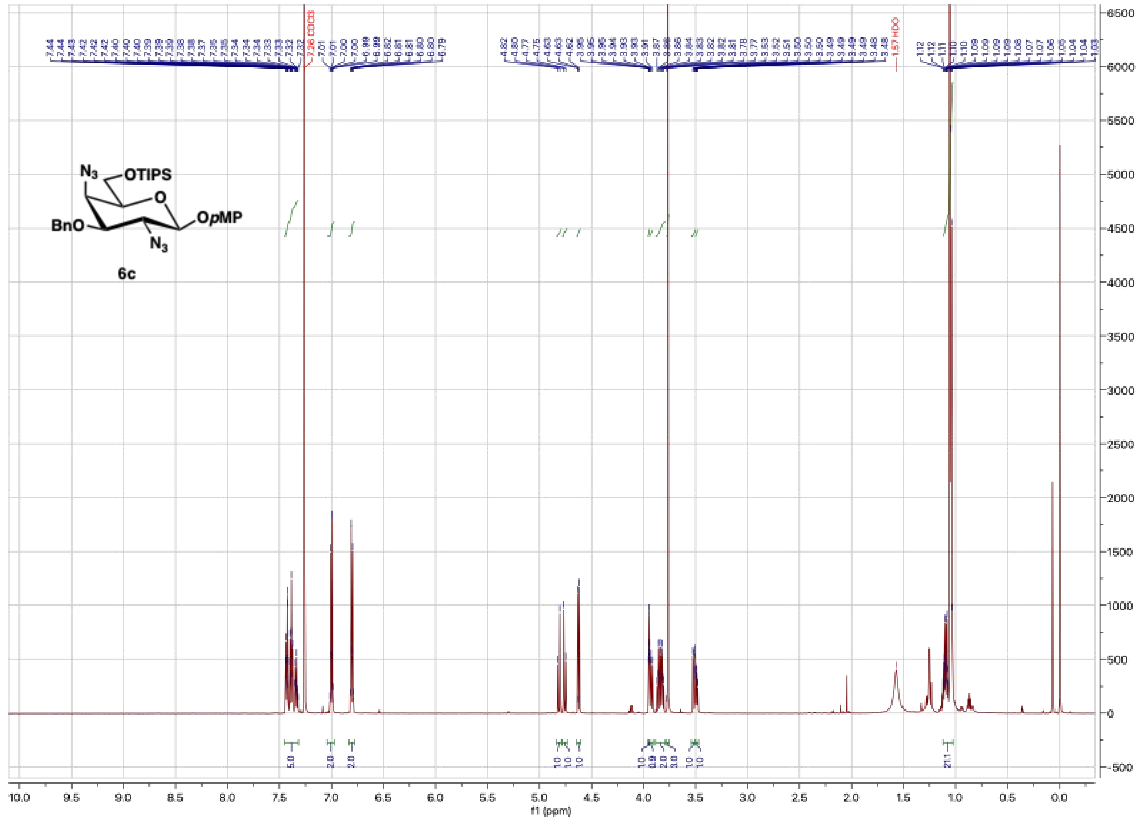

$^{13}C\{^1H\}$ NMR (151 MHz, $CDCl_3$)

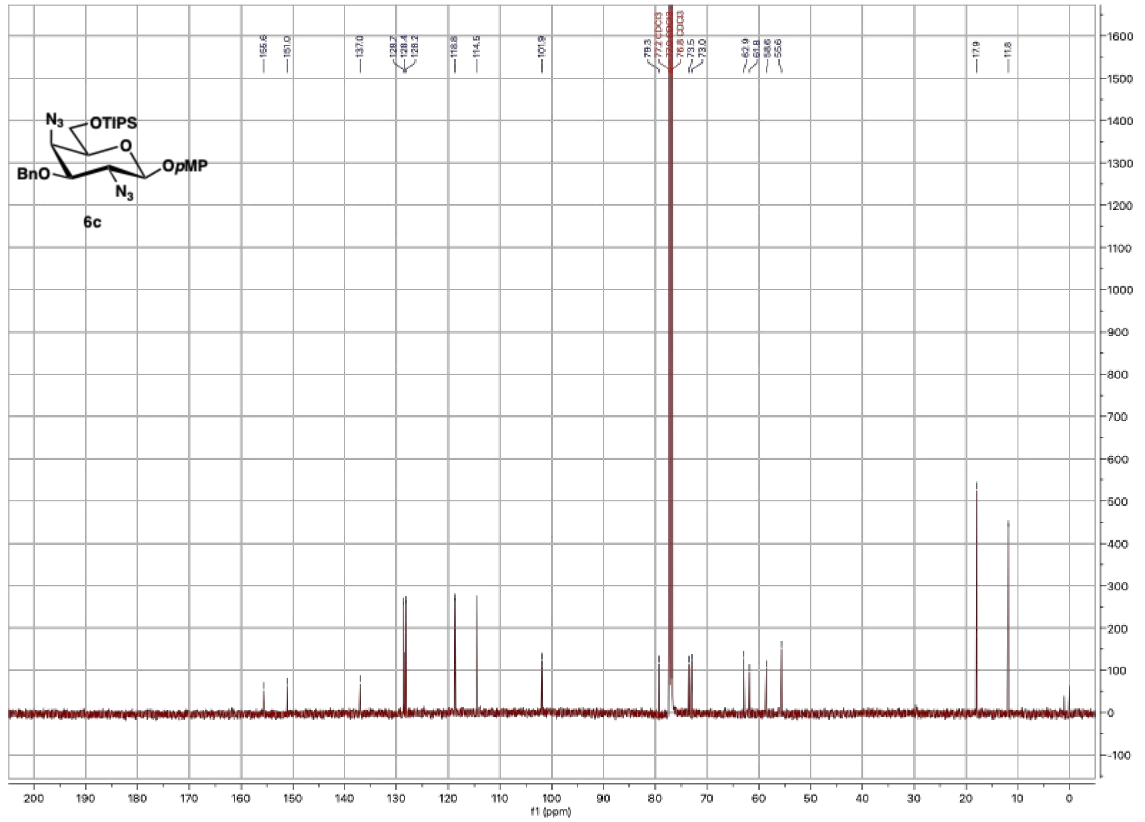

S-9

^{1}H NMR (400 MHz, $CDCl_3$)

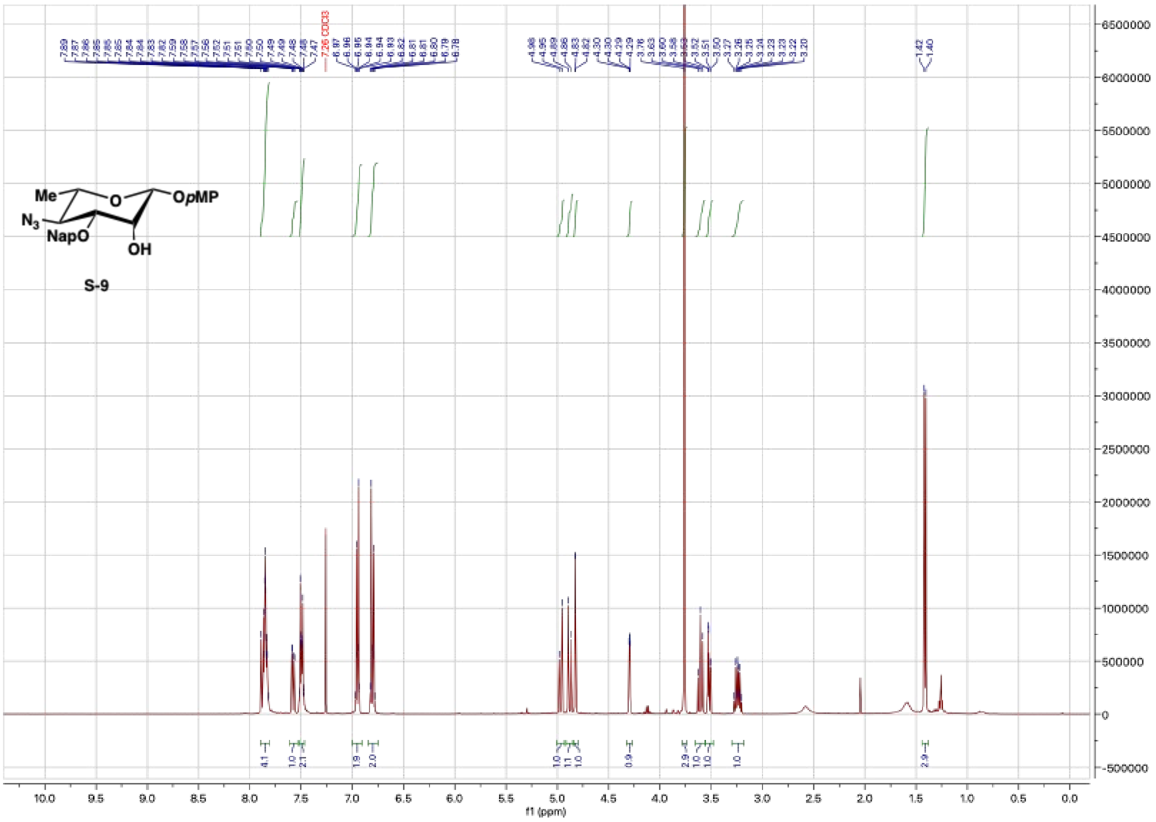

^{13}C{^{1}H} NMR (101 MHz, $CDCl_3$)

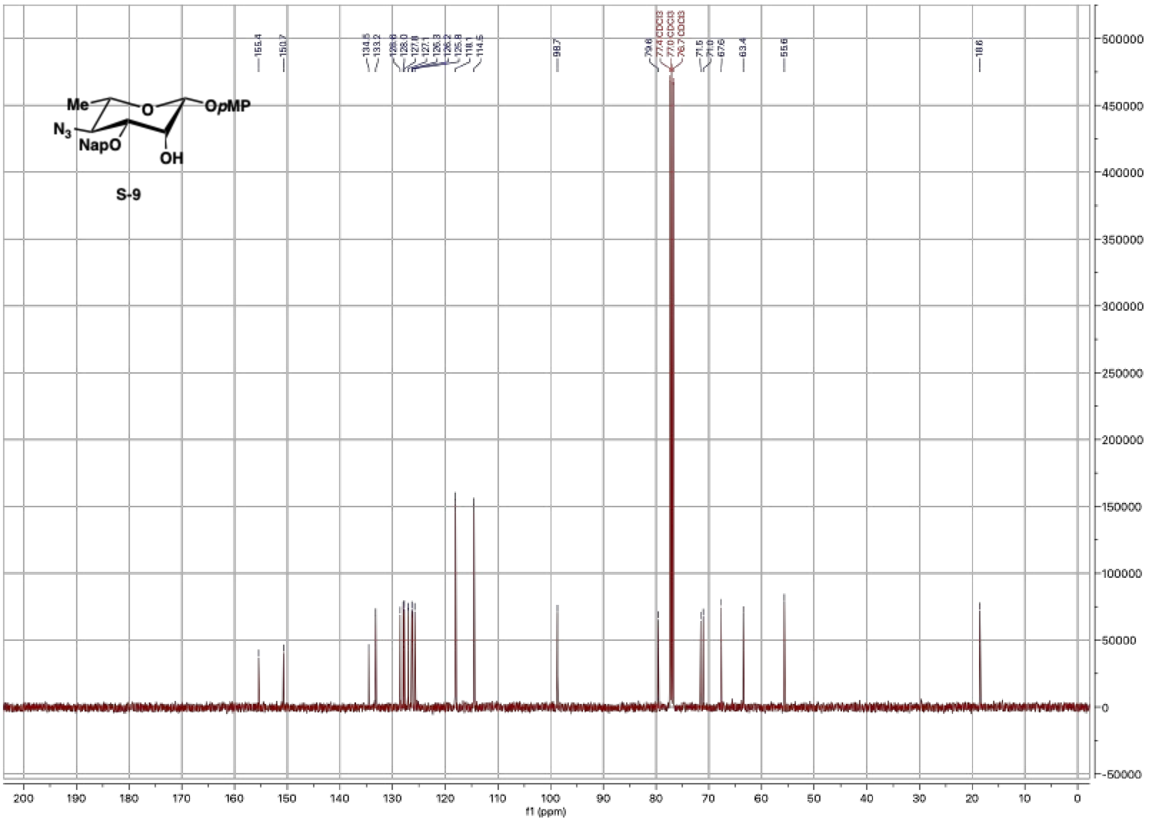

6d

^{1}H NMR (400 MHz, $CDCl_3$)

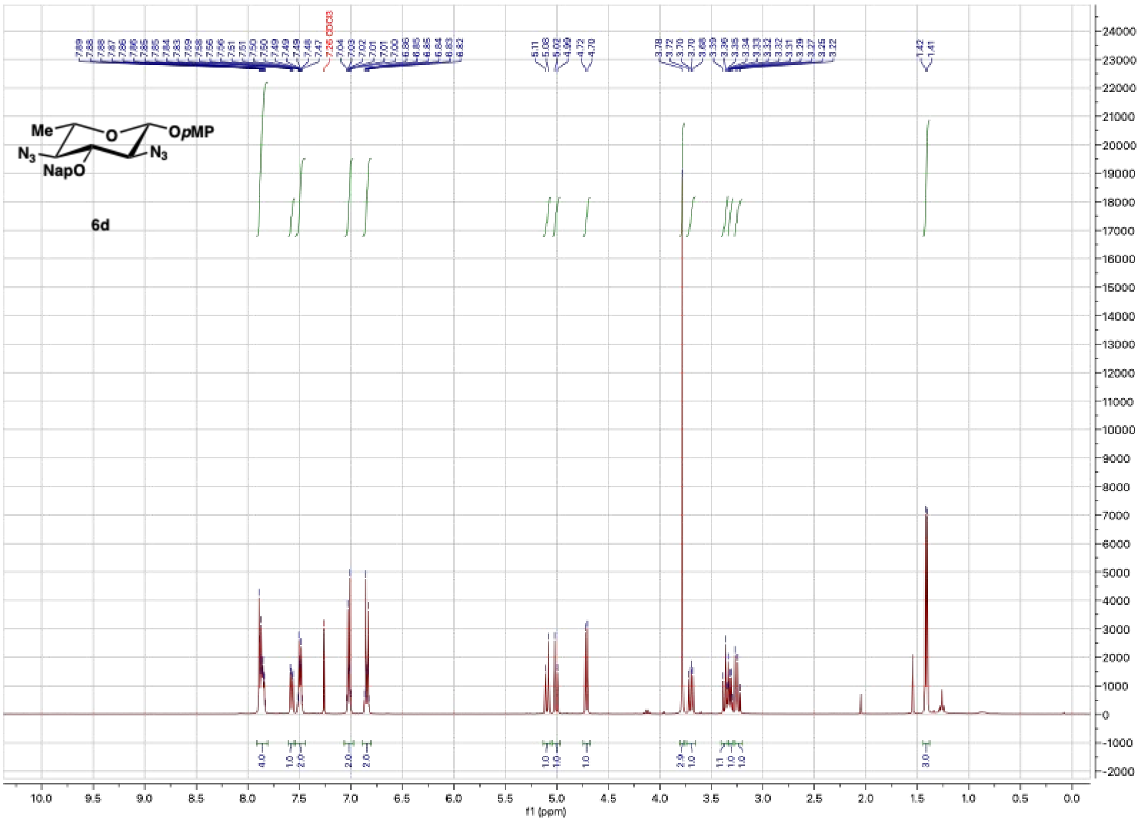

$^{13}C\{^{1}H\}$ NMR (101 MHz, $CDCl_3$)

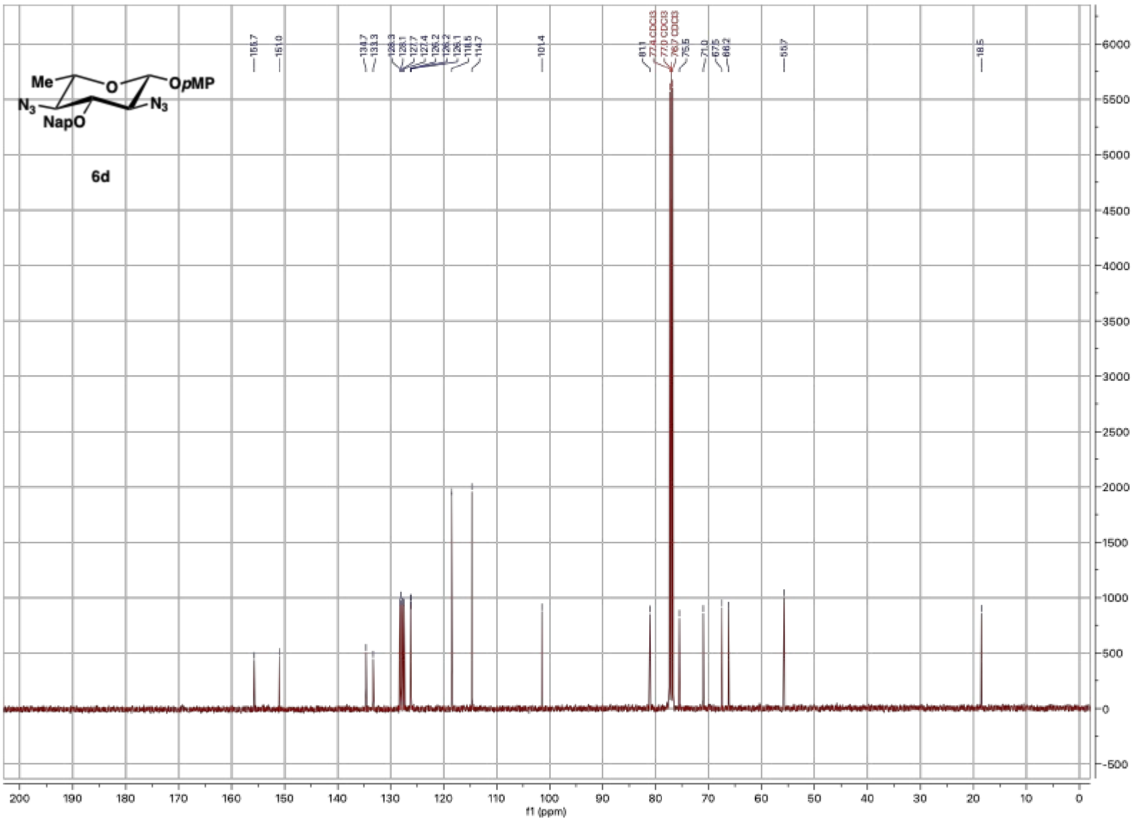

S-10

^{1}H NMR (600 MHz, $CDCl_3$)

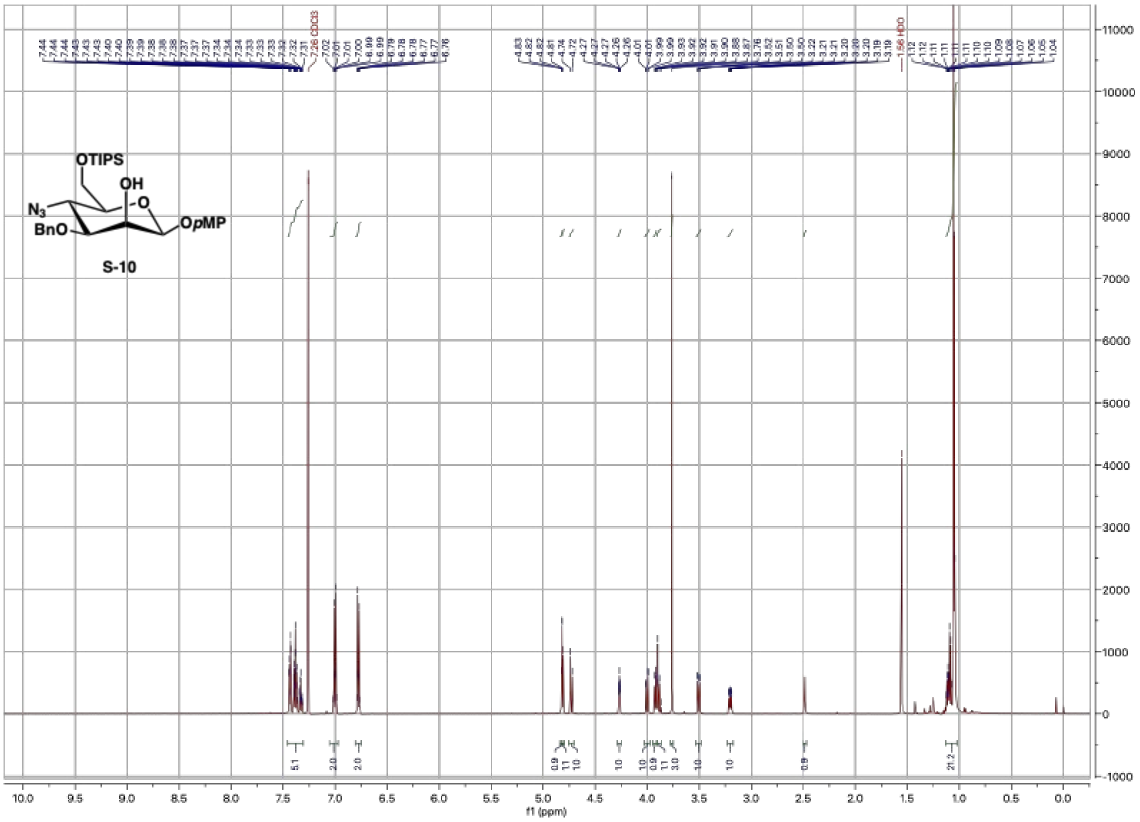

^{13}C{^{1}H} NMR (151 MHz, $CDCl_3$)

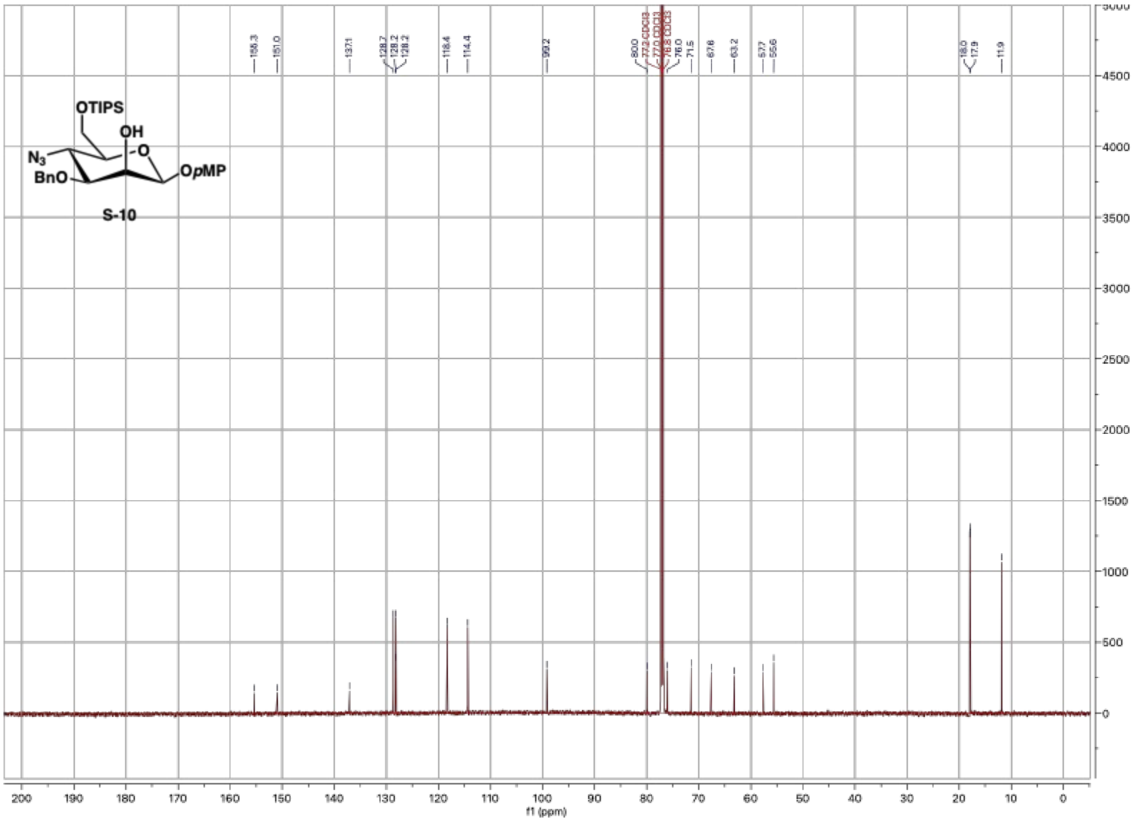

6e

^{1}H NMR (400 MHz, $CDCl_3$)

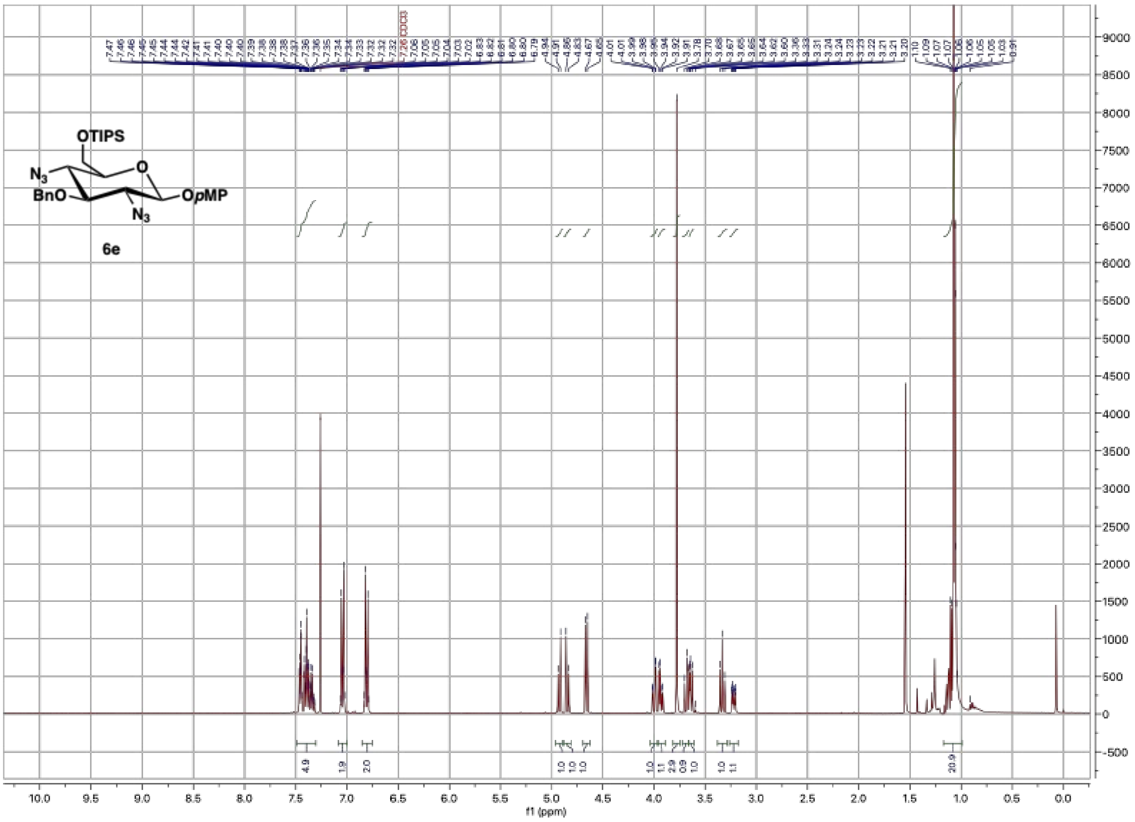

^{13}C{^{1}H} NMR (101 MHz, $CDCl_3$)

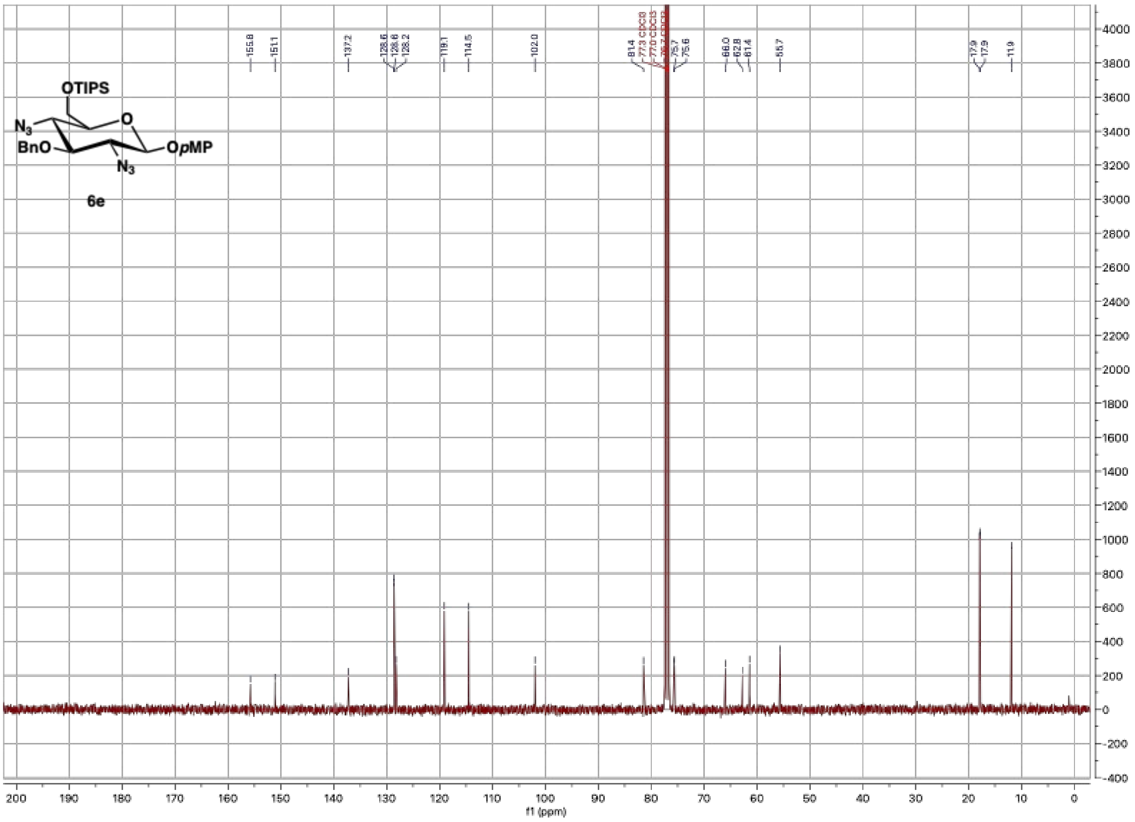

7

^{1}H NMR (400 MHz, $CDCl_3$)

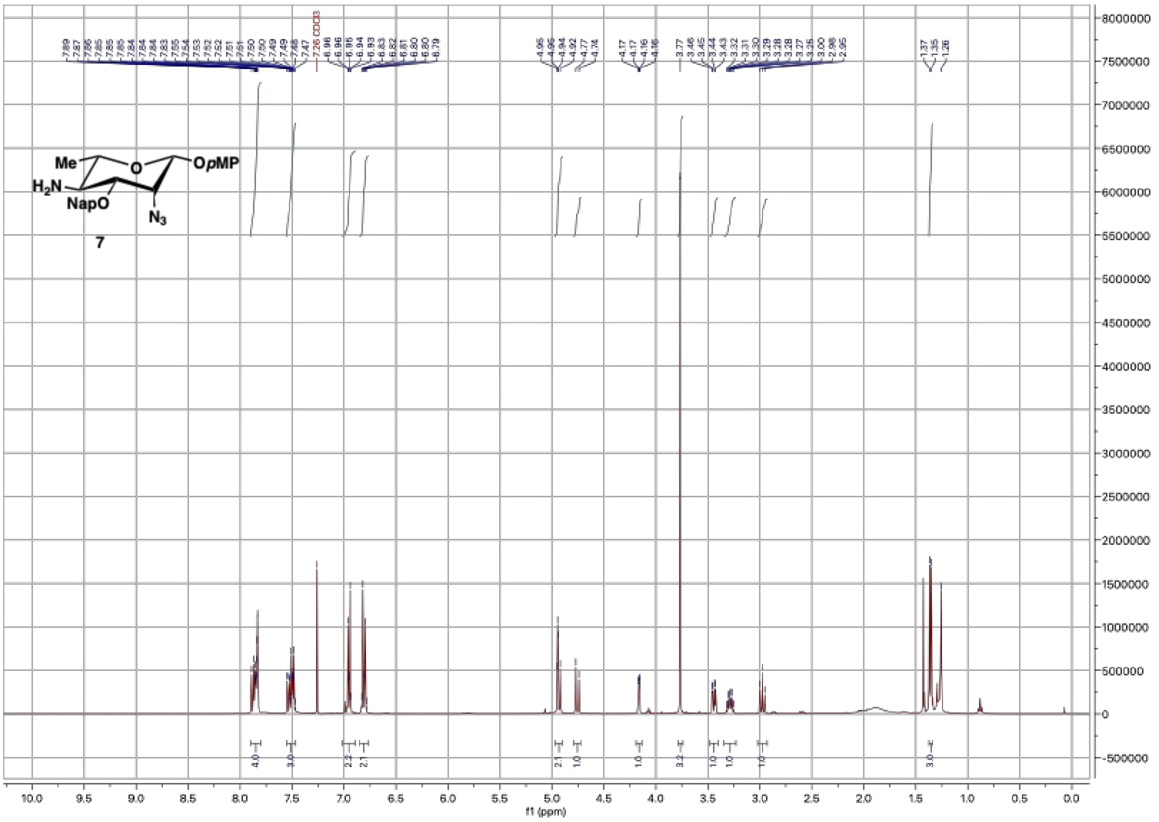

^{13}C{^{1}H} NMR (101 MHz, $CDCl_3$)

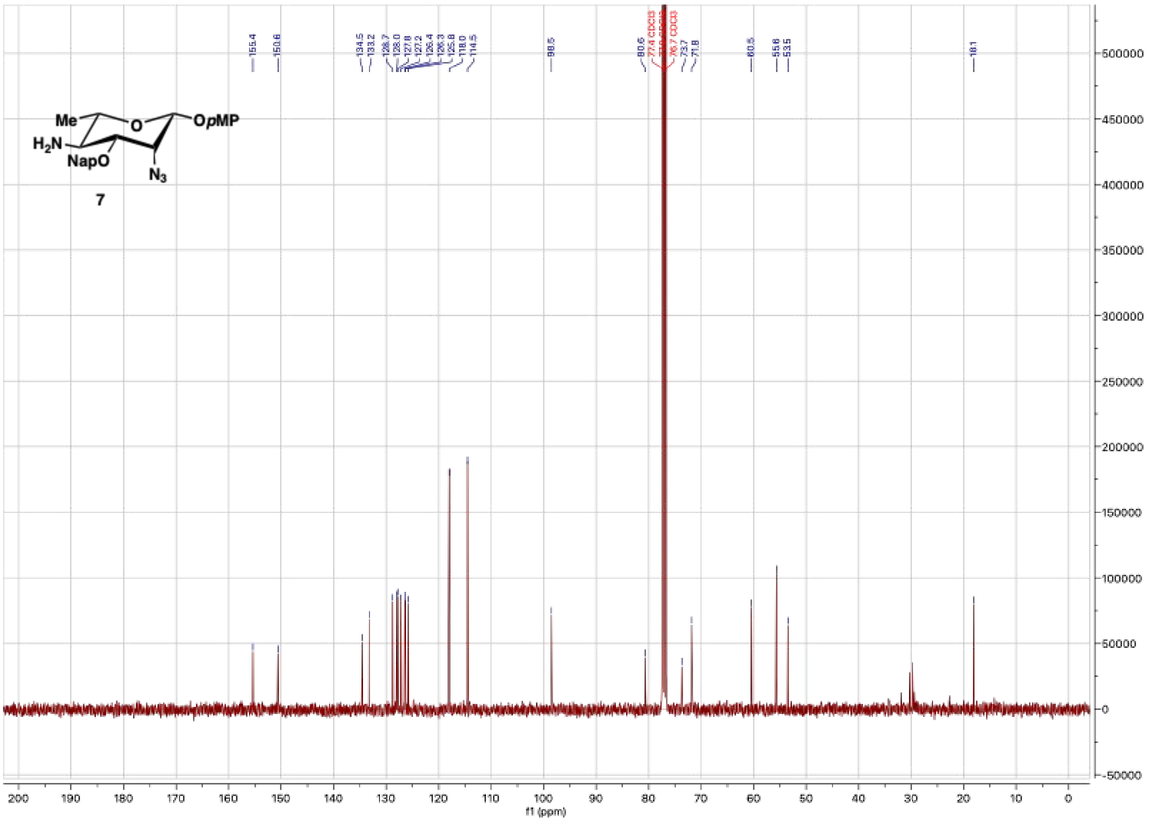

7a

^{1}H NMR (400 MHz, $CDCl_3$)

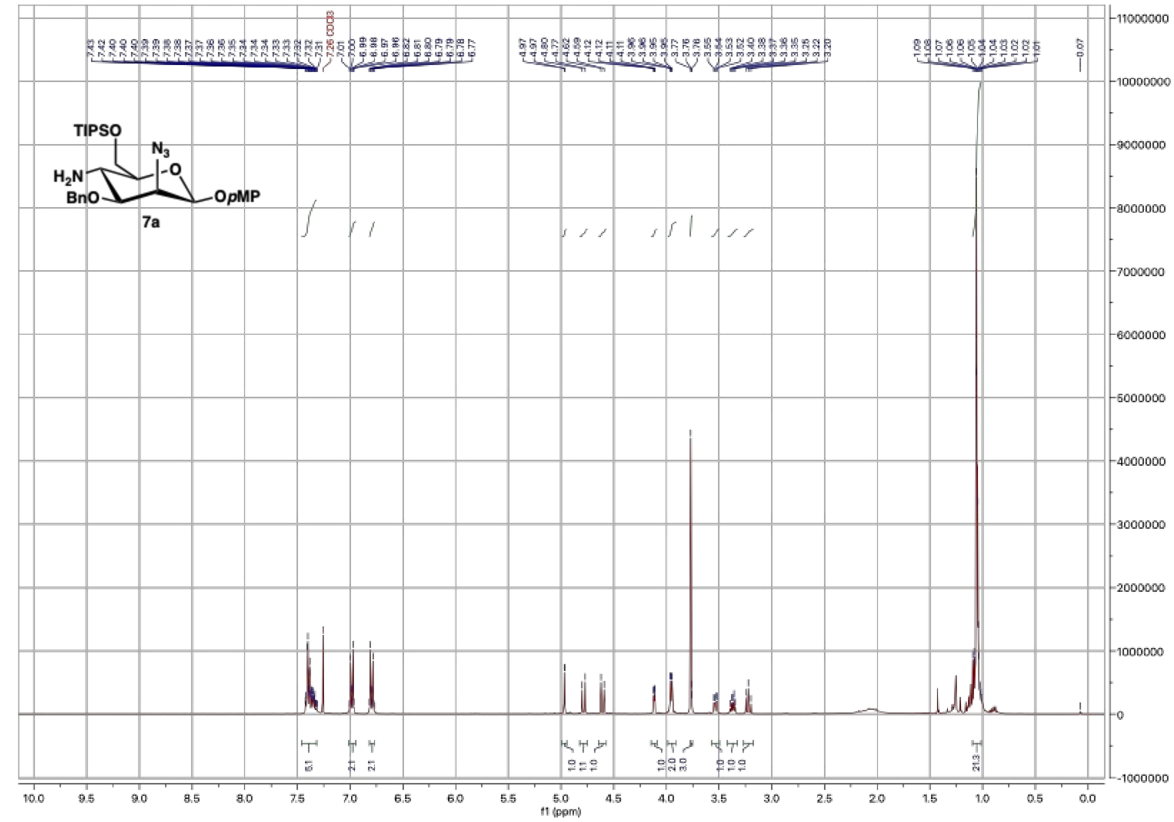

^{13}C{^{1}H} NMR (101 MHz, $CDCl_3$)

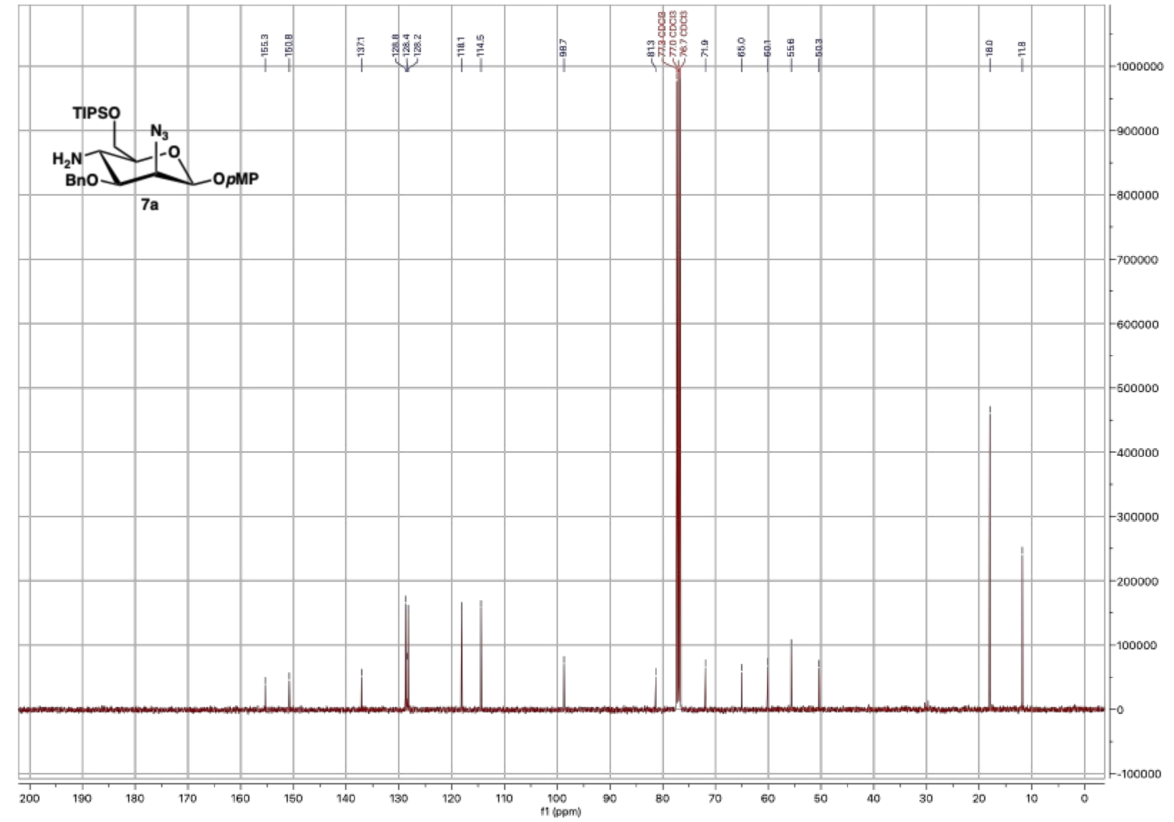

7b

^{1}H NMR (400 MHz, $CDCl_3$)

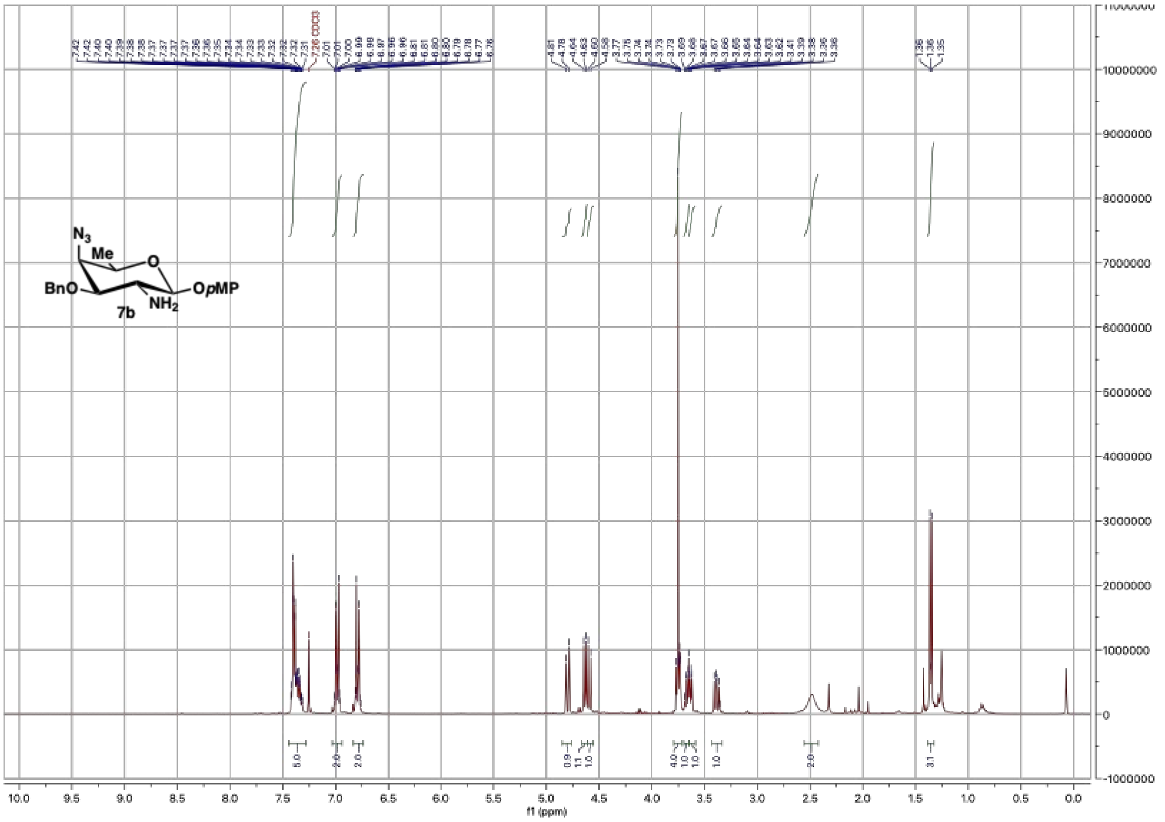

^{13}C{^{1}H} NMR (101 MHz, $CDCl_3$)

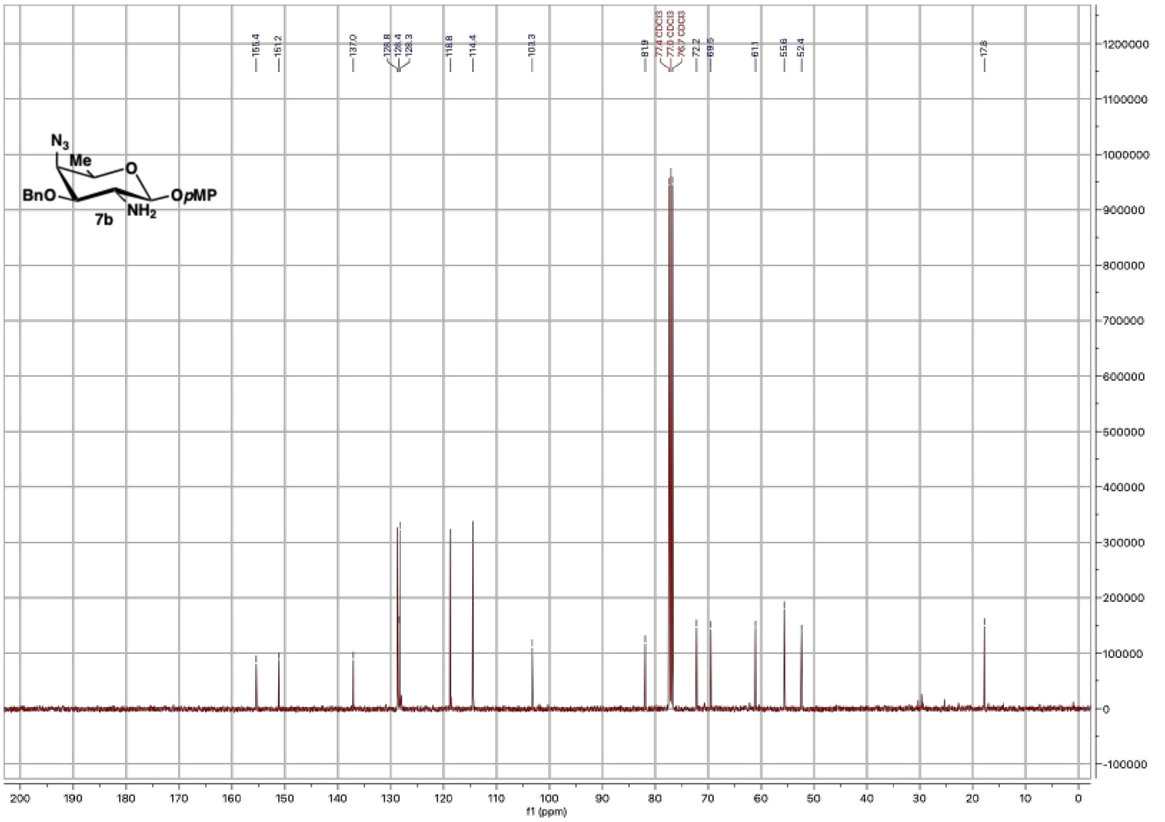

7c
^{1}H NMR (400 MHz, $CDCl_3$)

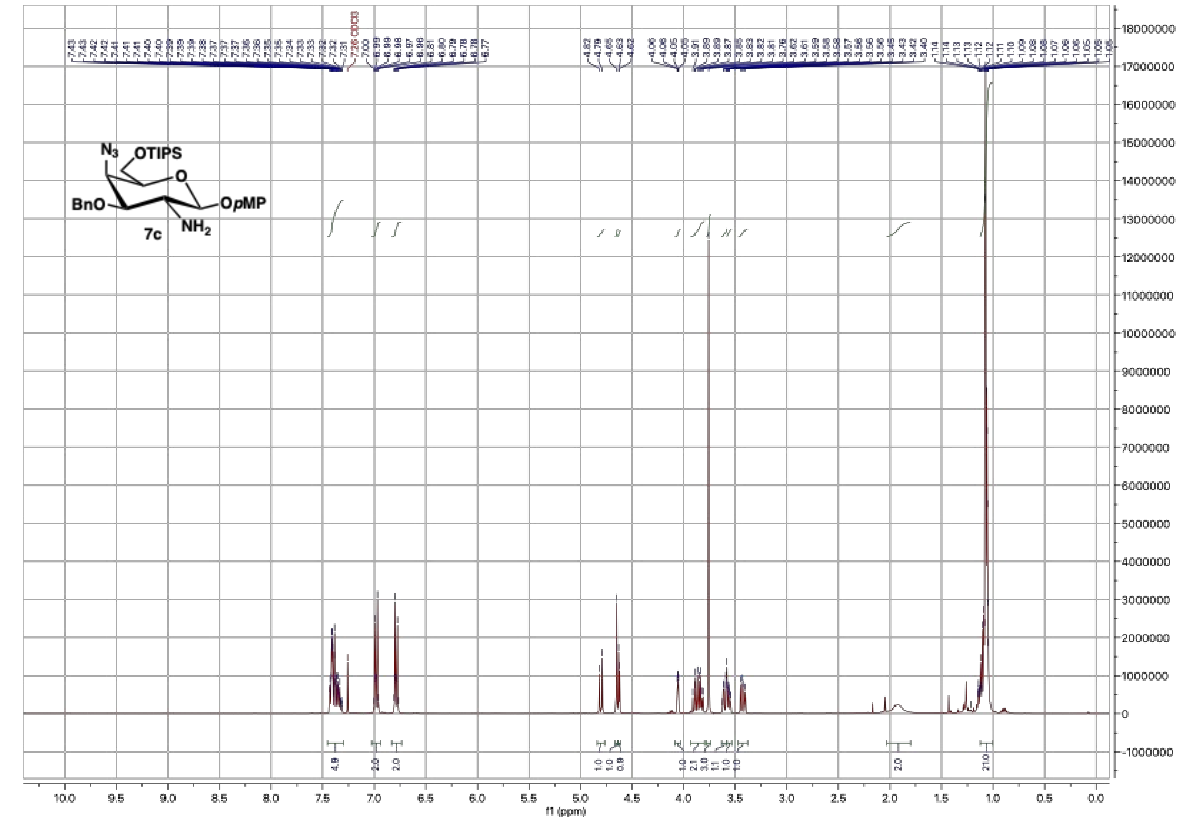

$^{13}C\{^{1}H\}$ NMR (101 MHz, $CDCl_3$)

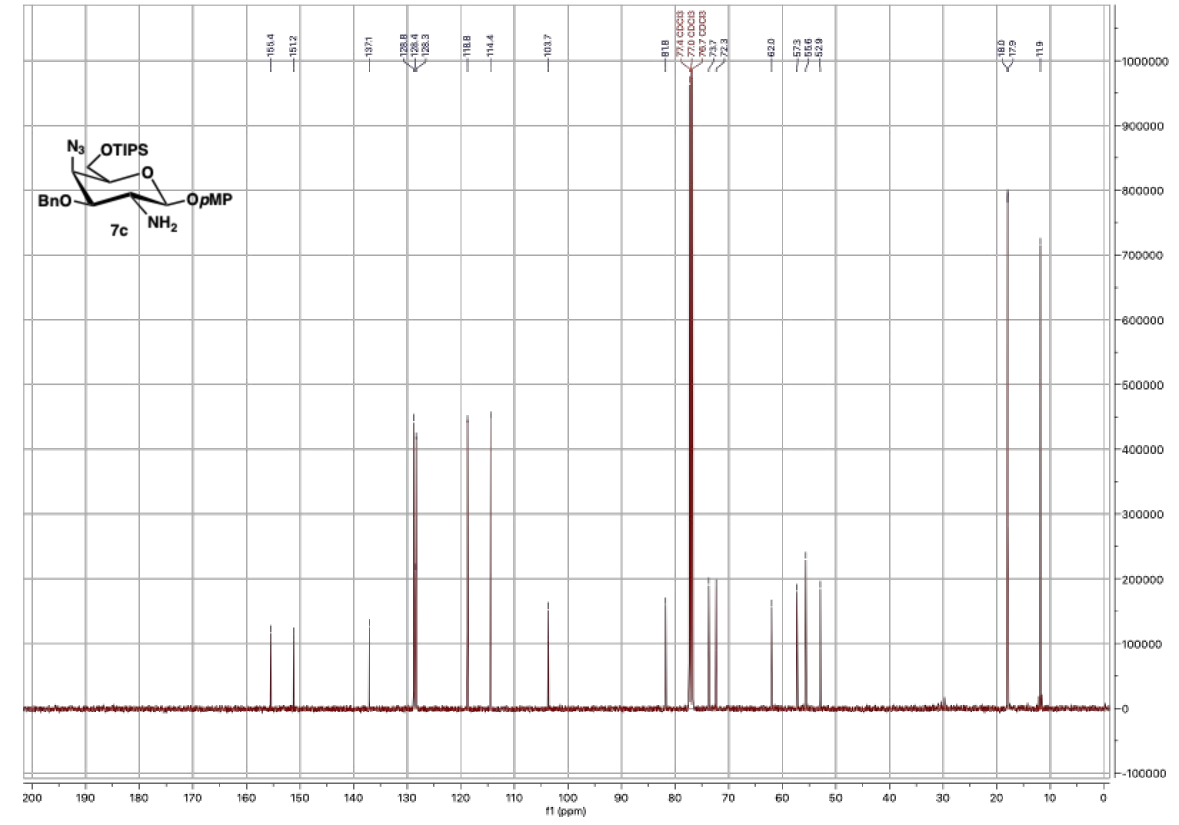

8

¹H NMR (400 MHz, $CDCl_3$)

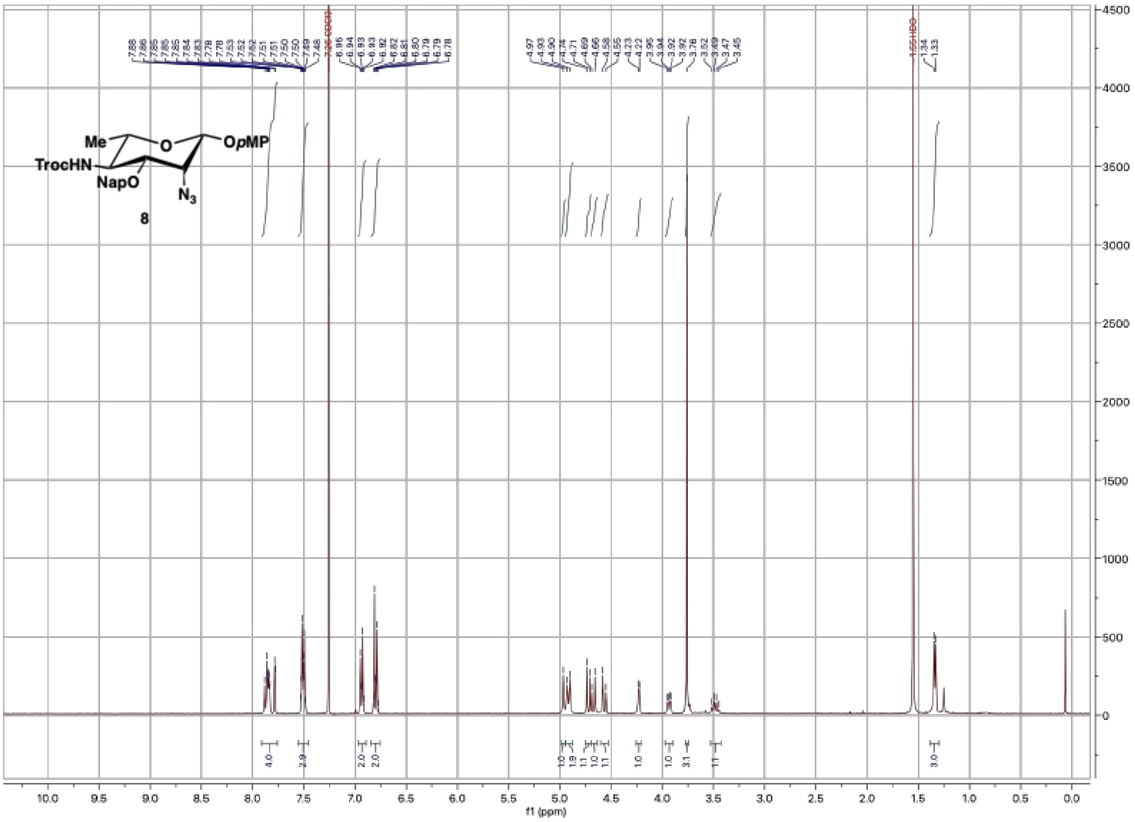

$^{13}C\{^1H\}$ NMR (101 MHz, $CDCl_3$)

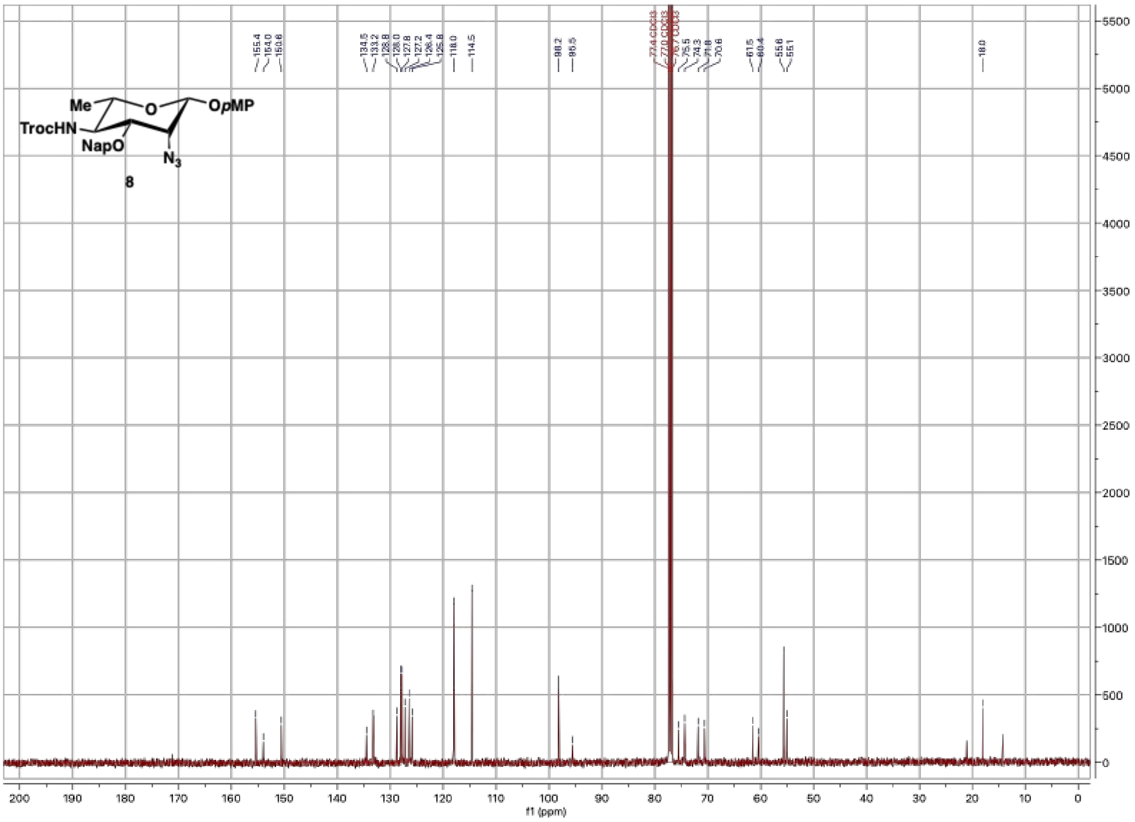

Supplement: Supplementary file 1 [file jo5c02433_si_001.pdf]
